# N-Acetyl-L-cysteine facilitates tendon repair and promotes the tenogenic differentiation of tendon stem/progenitor cells by enhancing the integrin α5/β1/PI3K/AKT signaling

**DOI:** 10.1186/s12860-022-00463-0

**Published:** 2023-01-05

**Authors:** Kang Lu, Mei Zhou, Liyuan Wang, Yang Wang, Hong Tang, Gang He, Huan Wang, Chuyue Tang, Jie He, Wei Wang, Kanglai Tang, Yunjiao Wang, Zhongliang Deng

**Affiliations:** 1 Department of Orthopedics-Spine Surgery Center, the Second Affiliated Hospital, Chongqing Medical University, No. 74 Linjiang Road, Yuzhong District, Chongqing, China; 2 Department of Orthopedics/Sports Medicine Center, State Key Laboratory of Trauma, Burn and Combined Injury, Southwest Hospital, Army Medical University, Third Military Medical University, No. 29, Yanzheng Street, Gaotan, Shapingba District, Chongqing, China

**Keywords:** NAC, TSPCs, Differentiation, Tendon injury, Rats

## Abstract

**Background:**

Tendon injury is associated with oxidative stress, leading to reactive oxygen species (ROS) production and inflammation. N-acetyl-L-cysteine (NAC) is a potent antioxidant. However, how NAC affects the biological functions of tendon stem/progenitor cells (TSPCs) and tendon repair has not been clarified.

**Method:**

The impacts of NAC on the viability, ROS production, and differentiation of TSPCs were determined with the cell counting kit-8, fluorescence staining, Western blotting, and immunofluorescence. The effect of NAC on gene transcription in TSPCs was analyzed by transcriptomes and bioinformatics and validated by Western blotting. The potential therapeutic effect of NAC on tendon repair was tested in a rat model of Achilles tendon injury.

**Results:**

Compared with the untreated control, treatment with 500 µM NAC greatly promoted the proliferation of TSPCs and significantly mitigated hydrogen peroxide-induced ROS production and cytotoxicity in vitro. NAC treatment significantly increased the relative protein expression of collagen type 1 alpha 1 (COL1A1), tenascin C (TNC), scleraxis (SCX), and tenomodulin (TNMD) in TPSCs. Bioinformatics analyses revealed that NAC modulated transcriptomes, particularly in the integrin-related phosphoinositide 3-kinase (PI3K)/AKT signaling, and Western blotting revealed that NAC enhanced integrin α5β1 expression and PI3K/AKT activation in TSPCs. Finally, NAC treatment mitigated the tendon injury, but enhanced the protein expression of SCX, TNC, TNMD, and COLIA1 in the injured tissue regions of the rats.

**Conclusion:**

NAC treatment promoted the survival and differentiation of TSPCs to facilitate tendon repair after tendon injury in rats. Thus, NAC may be valuable for the treatment of tendon injury.

**Supplementary Information:**

The online version contains supplementary material available at 10.1186/s12860-022-00463-0.

## Background

Tendon injuries are very common in orthopedics and sports medicine, and can cause severe pain, disability, and huge financial burden [1, 2]. Tendon injuries can happen suddenly (acute injury) or gradually (chronic injury), and may be caused by internal or external factors. The repair process is always accompanied by the formation of fibrovascular scars, fatty deposits, or ectopic ossification. The spontaneous healing process in injured tendons is generally difficult for the complete recovery of their function, and histological and mechanical characteristics [2–4]. Hence, the discovery of novel therapeutic reagents is of significance in promoting the functional recovery of the injured tendons.

A tendon injury can cause local inflammation that recruits inflammatory cell infiltration and promotes repair that attracts fibroblasts, tendon cells, and tendon stem/progenitor cells (TSPCs) to the lesions, leading to tendon regeneration and extracellular matrix (ECM) [1, 5, 6]. TSPCs were first reported in human and mouse tendons in 2007 and were subsequently confirmed in rat and rabbit tendons [7–9]. Similar to other stem cells, TSPCs have potent self-renewal ability and multi-differentiation potential because they can differentiate into tenocytes, adipocytes, chondrocytes, and osteocytes in optimal conditions [10, 11]. TSPCs are necessary for spontaneous healing to maintain their activity and regulate their differentiation, which are important for tendon repair [3, 10].

Oxidative stress (OS) can produce high levels of reactive oxygen species (ROS), which exhaust endogenous antioxidants [12]. During the process of repair after tendon injury, neovascular ingrowth at the site of injury provides excessive oxygen and blood supply, which can induce local OS reactions and inflammation to damage tenocytes and tenoblasts. ROS scavengers and antioxidants can reduce the non-tendon differentiation of tendon repair cells, tendon adhesion and inflammation in animal models [13–16]. Furthermore, OS can also lead to the occurrence of injury-related diseases, such as tendon degeneration, tear and fatigue fractures [17–19]. The application of antioxidants can alleviate local ROS accumulation and inflammation in injured tendons [16, 20, 21]. N-acetyl-L-cysteine (NAC) is the most common drug for neutralizing ROS to relieve OS and has been widely used in the respiratory and circulatory systems to relieve OS-related damage in cells [22–24]. Functionally, NAC can inhibit the generation of ROS and scavenge ROS because it can be deacetylated to form L-cysteine, which promotes the synthesis of endogenous glutathione to inhibit OS. In addition, NAC can also regulate cell metabolism, gene expression, and signal transduction, has anti-angiogenic and anti-apoptotic properties, and prevents DNA damage [24–26]. Protein phosphatase is a key molecule of signal transduction in cells. The redox state of cysteine residues in protein phosphatase is crucial for the biological behavior of cells. NAC, as a reducing agent, participates in the redox process of cells. Different from other reducing drugs, the NAC can be converted into cysteine in cells, which has a similar molecular structure to the cysteine residues in protein phosphatase. It can be inferred that NAC can simultaneously mediate biological behaviors, such as cell proliferation and differentiation [27–29] though regulating the phosphorylation state of related protein kinases. However, there are few reports on its biological effect of NAC on the differentiation of TSPCs in vitro and tendon repair in vivo.

This study explored the effects of NAC on the biological behaviors of TSPCs and the repair of injured tendon in vivo. Our results indicated that NAC effectively promoted the differentiation of TSPCs and the repair of injured tendons in rats. Therefore, NAC may be valuable for the treatment of tendon injury.

## Results

### NAC promotes the proliferation and survival of TSPCs

**Fig. 1:**
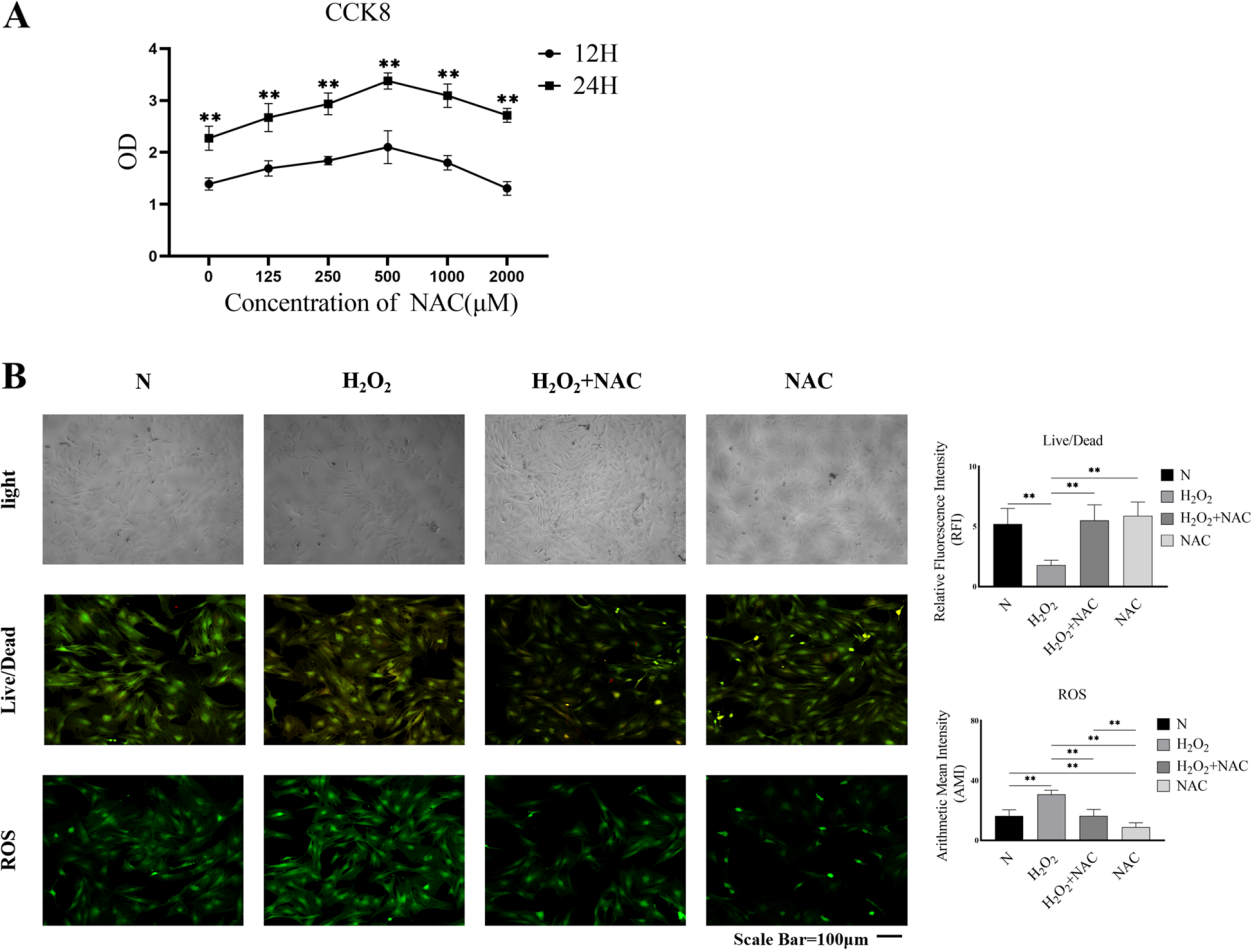
NAC treatment promotes the proliferation and survival of TSPCs. **A** CCK-8 analysis of the proliferation of TSPCs. TSPCs were treated in triplicate with, or without, the indicated concentrations of NAC for the indicated time periods and the viability of cells was analyzed by CCK-8 assays. **B** The morphology, live and dead cells and ROS production in TSPCs. TSPCs were cultured in triplicate in the presence or absence of the indicated compounds and their morphology was observed and the live/dead cells as well as their ROS production were characterized by fluorescent staining. Data are representative images or expressed as the mean ± SD of each group from three separate experiments. **P < 0.01

NAC is a potent scavenger of ROS. To determine the potential effect of NAC on the differentiation of TSPCs, we first examined the effect of NAC on the viability of TSPCs by CCK-8 assays. As shown in Fig. 1A, treatment with different doses of NAC promoted the proliferation of TSPCs and treatment with 500 µM NAC induced the highest effect in our experimental conditions. Next, we tested whether NAC could preserve the viability of TSPCs from H_2_O_2_ cytotoxicity. After the cells were treated with or without, H_2_O_2_ and/or NAC, the ratios of living to dead cells were reduced in the H_2_O_2_ group of cells but rescued by NAC, indicating that treatment with NAC preserved TSPCs from the H_2_O_2−_induced cytotoxicity (Fig. 1B). Analysis of ROS revealed that compared with control cells, the AMI of ROS signals in the H_2_O_2_ group of cells was significantly increased, which were abrogated by NAC treatment. In addition, treatment with NAC alone did not alter the ratios of living and dead cells but significantly reduced the ROS production in TSPCs. Hence, NAC treatment promoted the proliferation of TSPCs and protected them from the H_2_O_2_-induced cytotoxicity by reducing ROS production in vitro. Although the focus of this study was not on the mechanisms by which NAC regulated the proliferation and viability of TSPCs, the enhanced proliferation by NAC provided additional rationale for the application of NAC in tendon injuries.

### NAC treatment enhances the expression of tenogenic differentiation markers in TSPCs

**Fig. 2:**
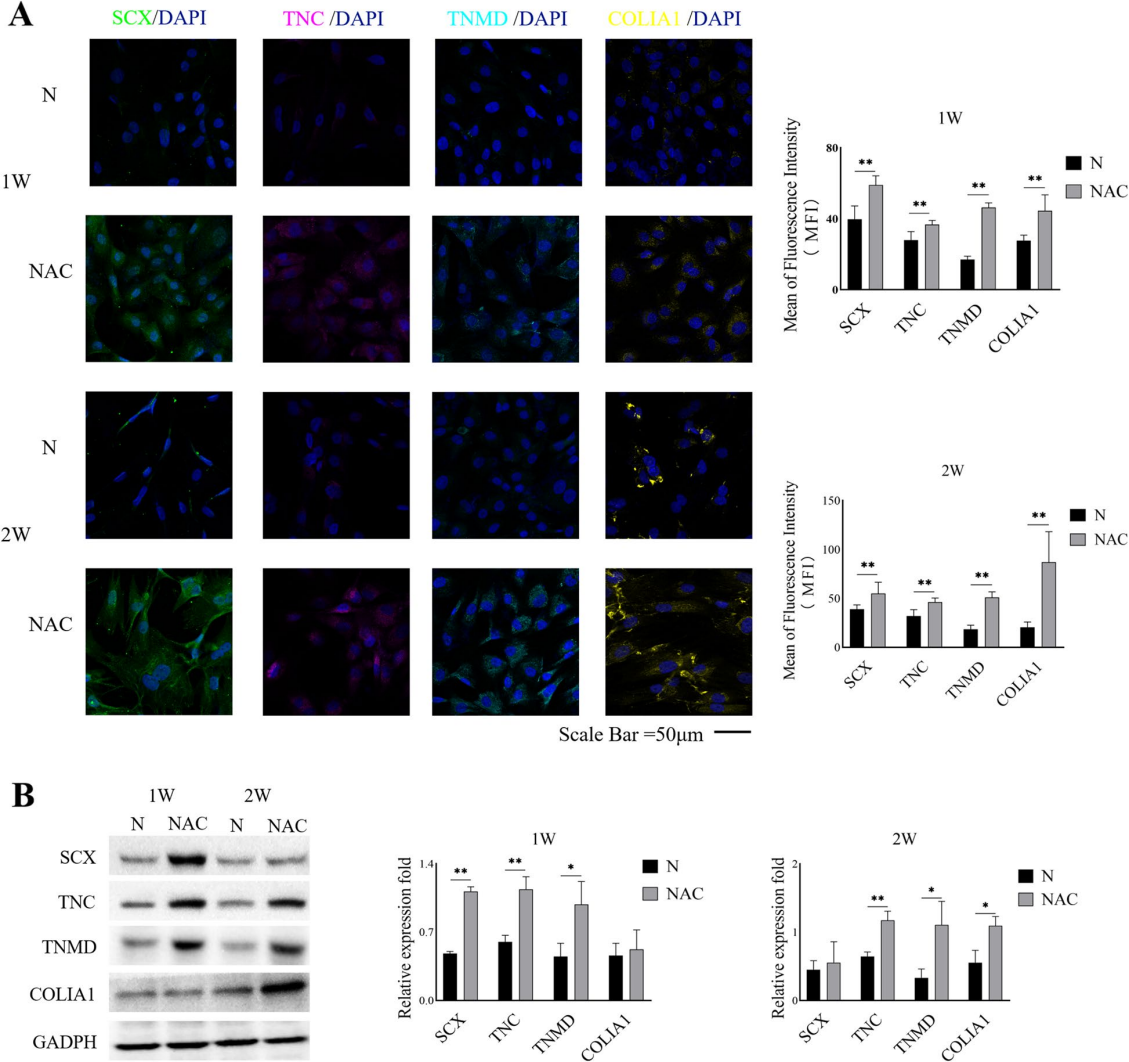
NAC treatment upregulates the expression of tenogenic differentiation factors in TSPCs. TSPCs were treated with, or without, NAC for 1 or 2 weeks and the levels of SCX, TNC, TNMD, and COLIA1 protein expression were determined by Western blotting and immunofluorescence. A. Immunofluorescence analysis of tenogenic differentiation factor expression in TSPCs. Data are representative images or expressed as the mean ± SD of each group from three separate experiments. B Western blot analysis of the expression of tenogenic differentiation factors in TSPCs. * *P* < 0.05; **P < 0.01

Immunofluorescence analyses revealed that the expression levels of SCX, TNC, TNMD, and COLIA1 in the NAC group were significantly higher than those in the N group at 1 and 2 weeks post culture (Fig. 2A). We assessed whether NAC could impact the differentiation of TSPCs by Western blot analysis. The results indicated that the expression of tenogenic differentiation markers of SCX, TNC, TNMD was significantly up-regulated at 1 week and the relative levels of TNC, TNMD, and COLIA1 expression significantly increased at 2 weeks in the NAC group compared to the N group of cells (Fig. 2B).

### NAC treatment alters the transcriptomics in TSPCs

**Fig. 3:**
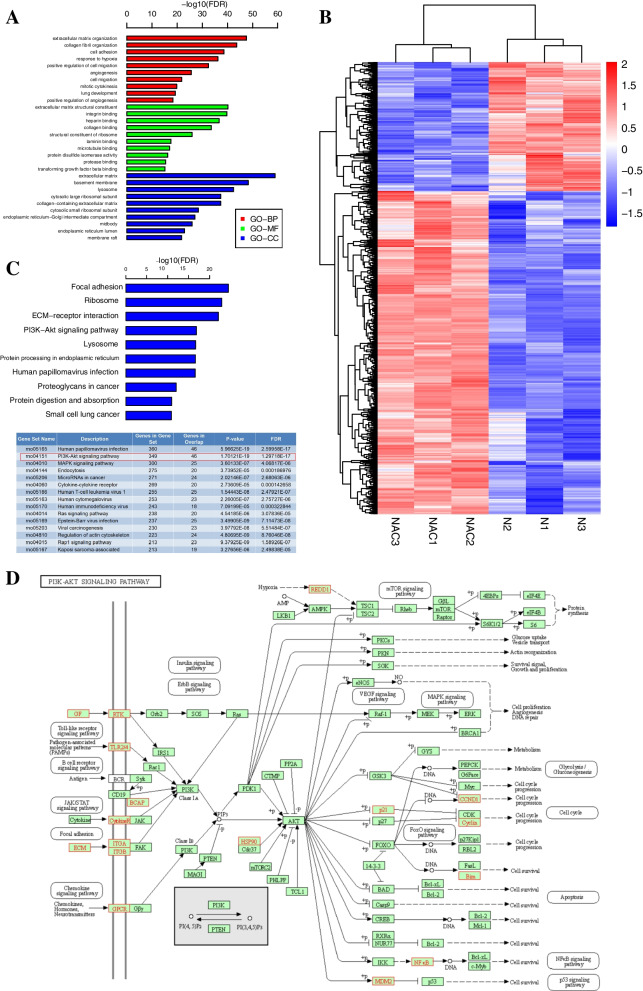
Bioinformatic analyses of DEGs induced by NAC. The gene expression bioinformatics analysis was performed using data obtained from RNA-seq of 2 group different cells after 7 days culturing. **A** GO analysis revealed that the DEGs centered on the main BP of ECM rganization and collagen fiber organization; the main MF of ECM structural constituent, integrin binding and collagen binding; the main CC of ECM and basement membrane. **B** The heatmap of the DEGs. **C** KEGG enrichment analysis indicated that DEGs focused on the PI3K/AKT signaling [30–32]. **D** A signaling network illustrated the DEGs concentrated in the integrin-related PI3K/AKT pathway

To understand how NAC treatment modulated the tenogenic differentiation of TSPCs, TSPCs were treated with or without NAC and their transcriptomics were analyzed. There were 1305 differentially expressed genes (DEGs), of which 932 were upregulated while 373 were downregulated in NAC-treated cells compared to the N group (Table S1). Gene Ontology (GO) analyses of the DEGs showed that the DEGs focused on the main biological process (BP) of ECM organization and collagen fiber organization; the main molecular function (MF) of ECM structural constituent, integrin binding and Col binding; and the main cellular component (CC) of the ECM and basement membrane (Fig. 3A,B). Kyoto Encyclopedia of Genes and Genomes analyses showed that the DEGs were enriched in the focal adhesion kinase and PI3K/AKT signaling pathways (Fig. 3C). In the PI3K/AKT pathway, the DEGs focused on the integrin-related downstream signaling (Fig. 3D).

### NAC treatment modulates the integrins α5β1 and PI3K/AKT signaling in TSPCs

**Fig. 4:**
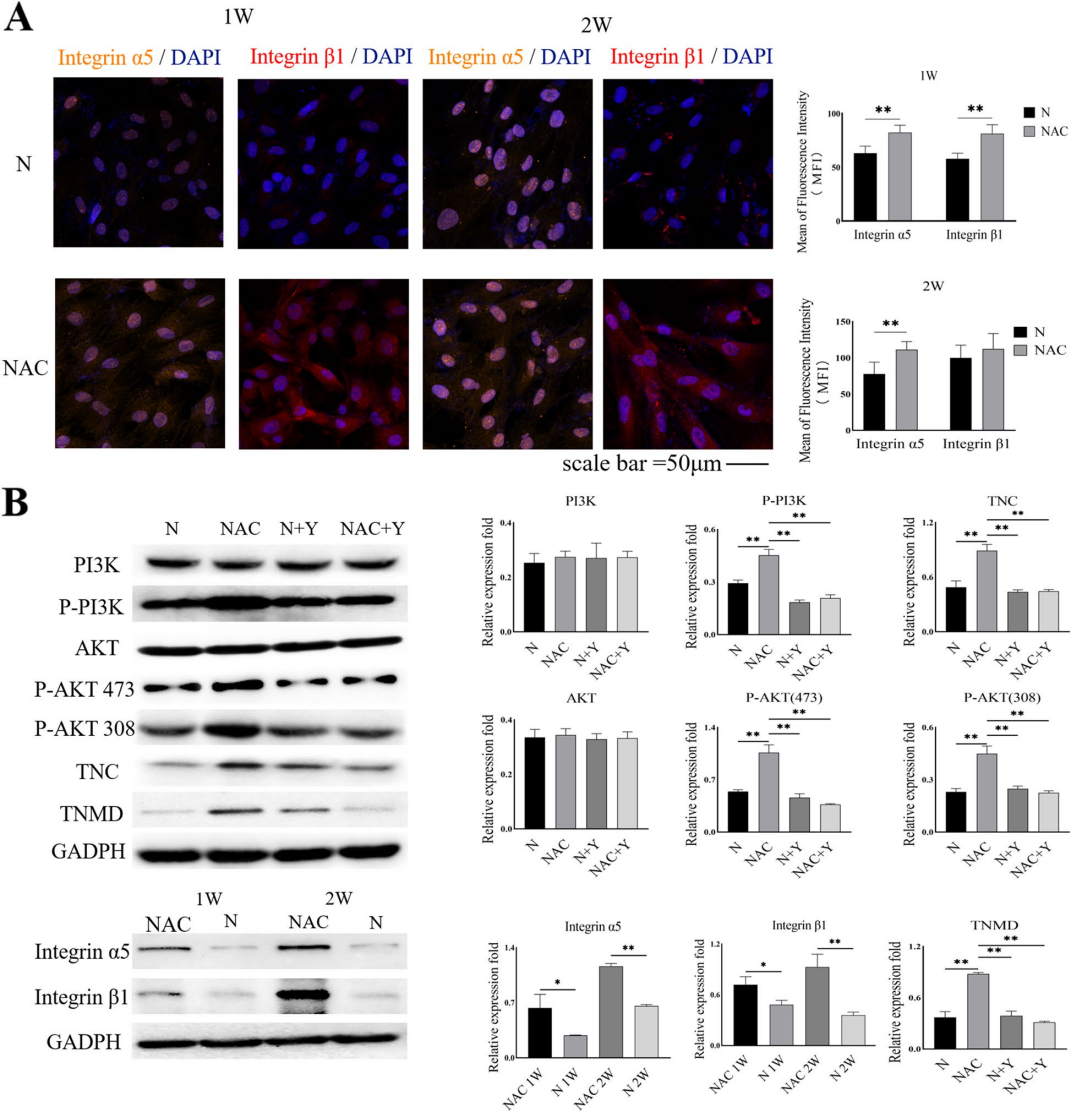
NAC upregulates integrin α5β1 protein expression and PI3K/AKT phosphorylation in TSPCs. TSPCs were treated in triplicate with, or without, NAC in the presence or absence of Ly294002 for 1 or 2 weeks, and the relative levels of integrin α5β1 protein expression and PI3K/AKT phosphorylation in TSPCs were determined by immunofluorescence and Western blotting. Data are representative images or expressed as the mean ± SD of each group from three separate experiments. * *P* < 0.05; **P < 0.01

We further validated whether NAC treatment modulated the integrin α5β1 and PI3K/AKT signaling in TSPCs by immunofluorescence. As shown in Fig. 4A, the MFI of integrin α5β1was significantly higher in the NAC group than in the N group. In addition, Western blot analysis indicated that NAC treatment upregulated the relative levels of integrin α5 and β1 in TSPCs compared to the N group. To further determine the importance of the PI3K/AKT signaling in NAC-modulated function, TSPCs were treated with, or without, NAC in the presence or absence of an PI3K/AKT inhibitor (LY294002), and the relative levels of PI3K/AKT phosphorylation and integrin α5β1 expression were analyzed by Western blotting. These results indicated that NAC treatment did not significantly alter the relative levels of PI3K and AKT expression, but significantly increased the relative levels of PI3K and AKT phosphorylation in TSPCs, which were abrogated by treatment with LY294002. In addition, treatment with LY294002 also mitigated the expression of TNC and TNMD in the NAC group of animals (Fig. 4B).

### NAC reduces OS and enhances tendon repair in rats following tendon injury

**Fig. 5:**
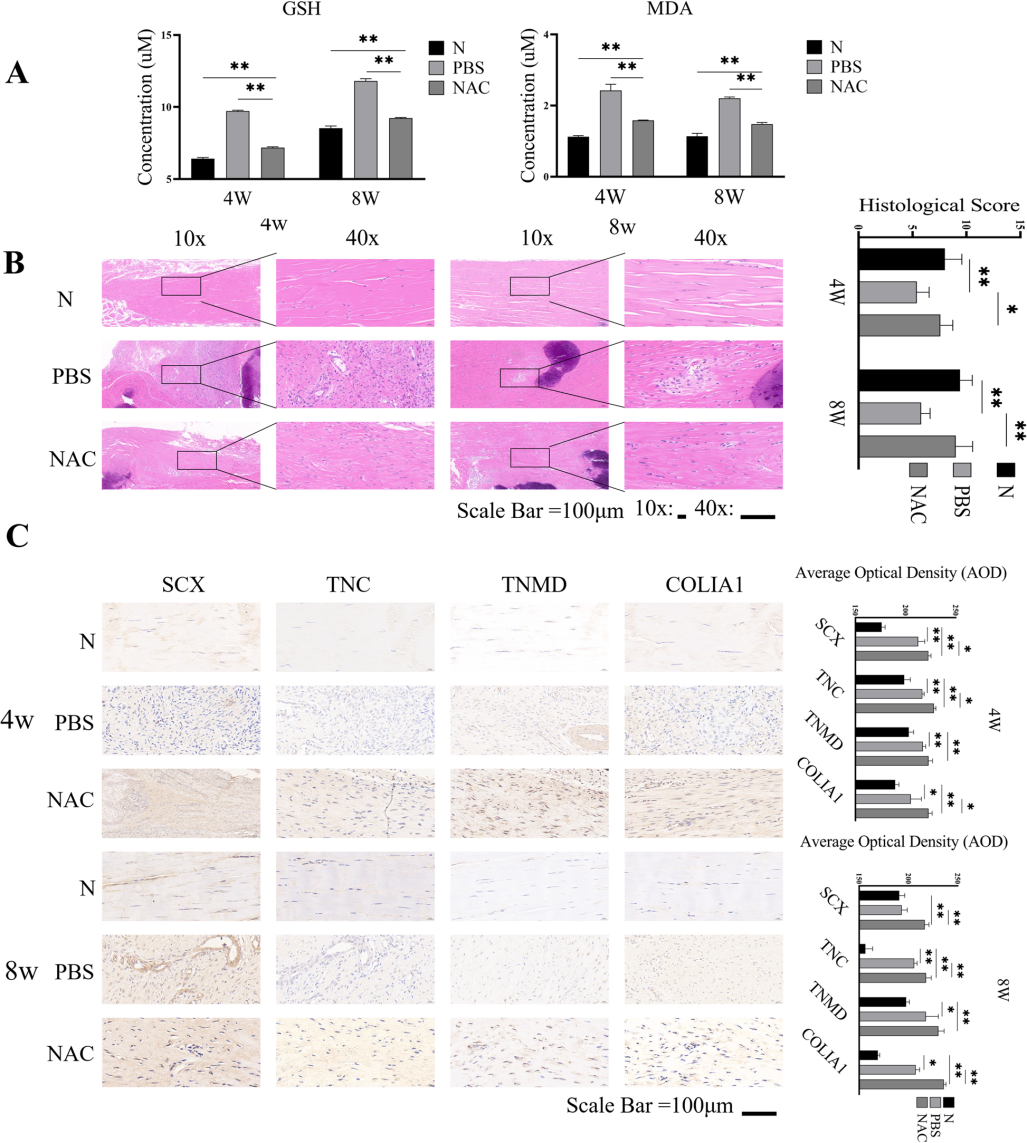
NAC treatment protects from tendon injury and promotes tenogenic differentiation of TSPCs in rats. Male Sprague-Dawley rats were randomized and subjected to the sham procedure (N group) or tendon injury, and the injured rats were randomized and treated with PBS (PBS group) or NAC (NAC group) for 4 or 8 weeks. Their tendon tissues were dissected and their tendon tissue sections were stained with hematoxylin and eosin or immunohistochemistry. Data are representative images or expressed as the mean ± SD of each group (*n* = 3 per time and group). **A** The contents of GSH and MDA in different groups of animals. **B** Histological analyses of tendon tissue morphology. **C** Immunohistochemistry analyses of the expression of indicated proteins in the tendon tissues of rats. *P < 0.05, **P < 0.01

As shown in Fig. 5A, GSH and MDA contents in NAC group were significantly lower than those in PBS group and higher than those in normal tissue (N group). In addition, we tested the potential effect of NAC treatment on tendon repair in rats. As shown in Fig. 5B, histological analyses showed that the collagen fibers were arranged in a disorderly manner in the PBS group, but NAC treatment preserved the continuity and orientation of collagen fibers in the NAC group. Quantitative analyses indicated that the histological scores of the NAC group were significantly higher than those of the PBS group. Immunohistochemical analyses revealed that the expression levels of SCX, TNC, TNMD, and COLIA1 in the tissues of the NAC group were significantly higher than those in the PBS group (Fig. 5C), suggesting that NAC may promote the differentiation of TSPCs and tendon repair in rats following tendon injury.

## Discussion

TSPCs have potent ability of self-renewal and multi-directional differentiation into tenocytes and tenoblasts as well as other types of cells, dependent on the environmental conditions, which is crucial for tendon healing and tendon tissue regeneration [10, 11, 33]. TSPCs exist in a low-blood supply and low-oxygen supply microenvironment [34, 35]. TSPCs were highly sensitive to ROS-induced cytotoxicity by reducing their capacity of self-renewal and differentiation [15, 35, 36]. NAC is a potent antioxidant and has been widely used in clinical practice to improve organ damage caused by oxidative stress [22–25] because NAC can scavenge ROS, minimizing oxidative stress-related cell damage [25, 37]. In this study, we found that treatment with NAC at a range of doses significantly promoted the proliferation of TSPCs in a dose-dependent manner and mitigated ROS production to preserve the survival of TSPCs in vitro.

The NAC can effectively eliminate accumulation of ROS and alleviate inflammation, which has application value in tendon repair. During the process of tendon injury and subsequent repair, tendon injury or lesion can promote local vascularization and cause OS and inflammation, leading to cell apoptosis and scar formation [13, 14, 17]. Injury-related OS and inflammation can also damage the survival and function of TSPCs, impairing tendon repair [29, 38] because the tenogenic differentiation of endogenous TSPCs is crucial for tendon repair after its injury. Hence, the control of OS and inflammation should be critical in promoting the tenogenic differentiation of TSPCs and tendon repair. We found that NAC treatment decreased the GSH and MDA contents in the injured tendon, reflecting that NAC treatment reduced OS and subsequently compensative GSH production following tendon injury. Furthermore, the NAC treatment significantly upregulated the relative levels of SCX, TNC, TNMD and COLIA1 protein expression in TSPCs and the tendon tissues of rats after tendon injury, highlighting the importance of NAC treatment in promoting the tenogenic differentiation of TSPCs in vitro and in vivo.

How did NAC promote the tenogenic differentiation of TSPCs? Redox reactions can modulate the signaling pathways to regulate the function of stem cells [12, 38, 39]. Previous studies have shown that integrins and related pathways are crucial for the dynamic regulation of cell differentiation and the phosphorylation/dephosphorylation status of a protein phosphatase in the signaling pathway, which are related to the oxidation/reduction status of cysteine residues on the protein phosphatase [40, 41]. To understand the molecular mechanisms underlying the action of NAC, we analyzed the impact of NAC on gene transcription of TSPCs by transcriptomes and bioinformatics. We found that the DEGs functioned for ECM remodeling and integrin-mediated cell adhesion. Indeed, treatment with NAC increased the expression of integrin α5β1 proteins in TSPCs. Integrin α5β1 acts as a membrane protein and sensor, and engagement of integrin α5β1 can activate the downstream signaling, including the PI3K/AKT pathway [42–45]. We found that the DEGs were significantly enriched in the PI3K/AKT signaling pathway and NAC treatment enhanced PI3K and AKT phosphorylation in TSPCs. NAC treatment also significantly upregulated the expression of TNC and TNMD proteins, which were abrogated by inhibitor of the PI3K/AKT signaling in the NAC group of TSPCs in vitro. These data indicate that NAC treatment can scavenge ROS indirectly enhance the integrin α5β1 and PI3K/AKT signaling pathways to promote the tenogenic differentiation of TSPCs. Supportively, the enhanced PI3K/AKT signaling can downregulate the classical apoptosis-related signaling [46, 47]. Furthermore, the PI3K/AKT signaling can promote the differentiation and fate of stem cells by modulating extracellular microbiomechanics [48–50]. Indeed, we did find that NAC-modulated DEGs were mainly responsible for cell adhesion and ECM synthesis in TSPCs. Therefore, we speculate that NAC may promote the tenogenic differentiation of TSPCs by changing cellular signals related to micro biomechanics and further activating the downstream integrin-PI3K/AKT signaling pathway. However, the precise mechanisms underlying the action of NAC need to be explored further.

The essence of tendon repair is the remodeling of collagen fibers in tendon tissues [51, 52]. To further test the therapeutic effect of NAC on tendon repair, we established a rat model of Achilles tendon injury and found that NAC treatment promoted the remodeling of collagen fibers and expression of key factors for tendon repair, mitigating the severity of tendon injury in rats. These data indicated that NAC preserved from injury-related tendon damages and promoted tendon repair after the tendon injury in rats. Thus, NAC may be a valuable reagent for the intervention of tendon injury. However, this study only preliminarily explored the role of several key protein molecules in the process of NAC-regulating TSPCs. The precise molecular mechanisms remain to be further explored in future researches, and the animal experimental model is also needed to be improved to clarify the changes in tendon biomechanics.

## Conclusion

In conclusion, our data indicated that NAC treatment promoted the survival and tenogenic differentiation of TSPCs and mitigated OS-mediated cytotoxicity against TSPCs by enhancing the integrin α5β1-PI3K/AKT signaling. NAC treatment also enhanced tendon collagen fiber remodeling to mitigate the severity of tendon injury and promote tendon repair in vivo. Therefore, NAC may be a potentially effective drug for the treatment of tendon injuries.

## Methods

### Cell viability assay

TSPCs were isolated from tendon tissues of three 8-week old male Sprague-Dawley (SD) rats as three independent biological samples and identified for their cell surface marker expression and multi-directional differentiation potential, as our previous study [33, 53]. All procedures and experiments were approved by the Ethics Committee of Army Medical University (Chongqing, China) and performed, according to the guidelines of the National Institutes of Health for the Care and Use of Laboratory Animals [53, 54]. The TSPCs were cultured in F12 complete media, which were changed every three days and the cells were passaged every xxx days. The TSPCs at passage 2 were harvested and used for subsequent experiments. The NAC was dissolved in sterile deionized water as a stock and diluted with F12 medium before cell culturing. TSPCs (5,000 cells/cm^2^) were treated in triplicate with vehicle as the N group, 125, 250, 500, 1000, or 2000 µM NAC in 96-well plates in F12 complete medium in a 5% CO_2_ incubator at 37 °C for 6 and 24 h. During the last 3 h of culture, 10% (v/v) of cell counting kit-8 (CCK-8) reagent was added to each well of the cultured TSPCs (Dojindo Molecular Technologies, Kumamoto, Japan). Subsequently, the absorbance of supernatants in each well was measured at 450 nm using a microplate reader (Model 680; Bio-Rad Laboratories, Hercules, CA, USA).

### ROS and live/dead cell staining

TSPCs (5000 cells/cm²) were cultured on a plastic petri dish and treated in duplicate with 500 µM NAC and/or 500 µM hydrogen peroxide (H_2_O_2_) for 12 h [55, 56]. The cells were stained for ROS (S0033M; Beyotime, Shanghai, China) and live/dead cells (BB4126; Bestbio, Nanjing, China), according to the manufacturer’s protocols. The biological experiments in this section were repeated for 3 times. The cells were examined and photographed under a laser scanning confocal microscope (LSM880; Zeiss, Wetzlar, Germany). The arithmetic means intensity (AMI) of the different fluorescent signals was analyzed using ZEN software (blue edition 2.3). Three different fluorescent images from each group were statistically analyzed.

### Bioinformatics analysis

The impact of NAC on gene transcription in TSPCs was analyzed by bioinformatics analyses, which were performed by Gene Biology (Shanghai, China). In brief, TSPCs were cultured in F12 complete medium (Group N) or F12 complete medium supplemented with 500 µM NAC (Group NAC) for 10 days. The TSPCs in both groups were harvested and washed with cold phosphate-buffered saline (PBS). The biological experiments were repeated for 3 times. Their total RNA was extracted using TRIzol reagent (Takara Bio, Shiga, Japan) and stored at -80 °C until transcriptomic analysis. The RNA samples were tested on 1% agarose gels for the potential RNA degradation and contamination. The RNA purity was measured using the Nano Photometer® spectrophotometer (IMPLEN, CA, USA). The RNA integrity was assessed using the RNA Nano 6000 Assay Kit in the Bioanalyzer 2100 system (Agilent Technologies, CA, USA). The feature Counts v1.5.0-p3 were used to count the read numbers mapped to each gene. The FPKM of each gene was calculated, based on the length of the gene and reads count mapped to this gene. Differential expression analysis of 3 conditions/groups (three biological replicates per condition) was performed using the DESeq2 R package (1.16.1). DESeq2 provides statistical routines for determining differential expression in digital gene expression data using a model, based on the negative binomial distribution. The resulting *P*-values were adjusted using the Benjamini and Hochberg’s approach for controlling the false discovery rate. Genes with an adjusted *P*-value < 0.05 by DESeq2 were assigned as differentially expressed genes (DEGs). Gene Ontology (GO) enrichment analysis of DEGs was performed using the cluster Profiler R package, in which gene length bias was corrected. GO terms with corrected P-value of < 0.05 were considered significantly enriched DEGs. The potential pathways of these DEGs were analyzed by KEGG using the cluster Profiler R package. The GATK2 (v3.7) software was used to perform SNP calling. Raw vcf files were filtered with the GATK standard filter method and other parameters (cluster:3; WindowSize:35; QD < 2.0 o; FS > 30.0; DP < 10) and SnpEff software was used to annotation for the Variablesite. The DEGs were analyzed by PPI in the STRING database to predict the potential protein-protein interactions.

### Immunofluorescence

TSPCs were cultured in medium alone (N group) or medium supplemented with 500 µM NAC (NAC group). The cells were fixed in 4% paraformaldehyde and washed three times with PBS. The fixed cells were incubated with primary antibodies overnight at 4 °C. After being washed, the bound antibodies were detected with secondary antibodies for 90 min at room temperature, followed by nuclearly staining with DAPI. The biological experiments in this section were repeated for 3 times. The primary antibodies from Bioss (Beijing, China) included anti-scleraxis (SCX, bs-12364R), anti-tenascin C (TNC, bs-1327R), anti-tenomodulin (TNMD, bs-7525R), anti-collagen type 1 alpha 1 (COLIA1, bs-10423R), anti-integrin α5 (bs-0567R), and anti-integrin β1 (bs-0486R). The secondary antibody was Cy3-Conjugated Affinipure Goat Anti-Rabbit IgG (SA00009-2; Proteintech, Wuhan, China). The concentrations of all antibodies were used, according to the manufacturer’s recommendation. All samples were examined under a laser scanning confocal microscope. The mean fluorescence intensity (MFI) in each field was analyzed using ZEN software. Three different fluorescent images from each group were statistically analyzed.

### Western blot analysis

TSPCs were cultured in medium alone (N group) or with 500 µM NAC (NAC group) in the presence or absence of LY294002 (Bioss), an inhibitor of the phosphoinositide 3-kinase (PI3K)/AKT signaling, as the intervention group for 1 and 2 weeks (the same medium for each group of cells was replaced every three days). The biological experiments were repeated for 3 times. TSPCs in the N or NAC group were lysed in lysis buffer (50 mmol/L Tris-HCl, pH 8.0, 1 mmol/L EDTA, 1% Triton X‐100, 0.5% sodium deoxycholate, 0.1% sodium dodecyl sulfate (SDS), 150 mmol/L NaCl) containing proteinase inhibitors (Thermo Fisher Scientific, Rockford, IL, USA). Their protein concentrations were determined using the BCA Protein Assay Kit (Thermo Fisher Scientific). The cell lysates (30 µg/lane) were separated by SDS–polyacrylamide gel electrophoresis and transferred onto polyvinylidene difluoride membranes. After blocking in 5% non-fat milk in TBST, the membranes were incubated with the following primary antibodies: anti-SCX (1:1000, ab58655; Abcam, Cambridge, UK), anti-TNC (1:1000, ab108930; Abcam), anti-TNMD (1:1000, ab203676; Abcam), anti-COLIA1 (1:1000, ab6308; Abcam), anti-AKT (1:1000, 9272 S; Cell Signaling Technology [CST], Danvers, MA, USA), anti-phospho-AKT (Ser473, 1:1000, 9271 S; CST), anti-phospho-AKT (Thr308, 1:1000, 9275 S; CST), anti-PIK3CA (1:1000, bs-2067R; Bioss), anti-phospho-PI3KCA (1:1000, bs-5570R; Bioss), anti-integrin alpha 5 (1:1000, bs-0567R; Bioss), anti-integrin beta 1 (1:1000, bs-0486R; Bioss), and anti-GAPDH (1:1000, ab8245; Abcam). After being washed, the membranes were incubated with horseradish peroxidase (HRP)-conjugated secondary antibody (10,285‐1‐AP, 1:2000; Proteintech, Wuhan, China) and visualized by enhanced chemiluminescence. The results were analyzed by ImageJ software.

### Achilles tendon injury and repair in rats

The animal experiments were approved by the Ethics Committee of the Army Medical University (Chongqing, China) and performed according to the guidelines of the National Institutes of Health for the Care and Use of Laboratory Animals. Male Sprague-Dawley rats (8 weeks, 180–200 g) were obtained from Southwest Hospital Animal Center (Chongqing, China). The rats in individual groups (n = 3 per group) were anesthetized intraperitoneally with pentobarbital. Their right hind limb was shaved, disinfected with povidone iodine and subjected to a 2-cm longitudinal incision to expose the Achilles tendon [57]. Subsequently, the rats in the PBS and NAC groups were induced for a transection injury of the medial gastrocnemius tendon (transected for two-thirds of the Achilles tendon) and sutured using 5 − 0 absorbable sutures. The rats were randomized and injected with 0.2 mL PBS or 500 µM NAC solution at the injury site every 3 days beginning on day 8 post-injury to determine the remodeling effect of NAC on tendon fibers during the repair process. The N group of rats received the sham procedure with PBS injection. The animals were euthanized at 4 or 8 weeks post-induction and their relevant tissues were dissected for subsequent experiments.

### Measurements of GSH and MDA

The tissue samples were frozen in liquid nitrogen and powdered samples (10 mg each) were mixed with 30 µl of protein-removal reagent, and centrifuged at 10,000 g, 4 ° C for 10 min. Their supernatants were collected for the determination of reduced glutathione (GSH, S0053, Beyotime, Shanghai, China) and malonic dialdehyde (MDA, S0131S, Beyotime, Shanghai, China) using specific kits, following the manufacturer’s protocols.

### Histology

The dissected tissue specimens were fixed in 10% formaldehyde overnight and paraffin-embedded. The tissue Sect. (5 μm) were stained with hematoxylin and eosin and imaged under an optical microscope (Olympus, Tokyo, Japan). The biological experiments in this section were repeated for 3 times. The pathological changes in three fields were evaluated using the modified histological scoring system (Table S2) [33].

### Immunohistochemistry

The tissue Sect. (5 μm) were dewaxed, rehydrated and regularly stained with antibodies to evaluate the expression of tendon repair markers by immunohistochemistry. The biological experiments were repeated for 3 times. The primary antibodies were anti-TNC (1:100, bs-1039R), anti-ColI (bs-10423R), anti-SCXA (bs-12364R), and anti-TNMD (bs-7525R) from Bioss. The HRP-conjugated goat anti-rabbit antibodies were used as secondary antibodies (1:200, GB23303; Servicebio, Wuhan, China). The immunohistochemistry signals were imaged under an inverted fluorescence microscope (IX71; Olympus) and the average optical density (AOD) was measured using ImageJ software.

#### Statistical analyses

All values are presented as the mean ± standard deviation (SD) from three separate experiments with at least three biological samples. Quantitative data were analyzed by one way analysis of variance and post hoc Tukey test and the difference between groups was tested by independent samples *t*-test using SPSS 26.0 software. A *P*-value of < 0.05 was considered statistically significant. 

## Supplementary Information

**Additional file 1.**

**Additional file 2.** **TableS2.** Histological scoring.

**Additional file 3.**

**Additional file 4.**

## Data Availability

The data that support the findings from this study are available on request from the corresponding authors. The data are not publicly available due to privacy or ethical restrictions.

## Abbreviations

ROS: Reactive Oxygen Species
NAC: N-acetyl-L-cysteine
TSPCs: Tendon Stem/Progenitor Cells
COL1A1: Collagen Type 1 Alpha 1
TNC: Tenascin C
SCX: Scleraxis
TNMD: Tenomodulin
PI3K: Phosphoinositide 3-kinase
ECM: Extracellular Matrix
OS: Oxidative Stress
ROS: Reactive Oxygen Species
CCK-8: Cell Counting Kit-8
H_2_O_2_: Hydrogen Peroxide
AMI: Arithmetic Means Intensity
PBS: Phosphate-Buffered Saline
MFI: Mean Fluorescence Intensity
SDS: Sodium Dodecyl Sulfate
HRP: Horseradish Peroxidase
SD: Standard Deviation
DEGs: Differentially expressed genes
GO: Gene Ontology
BP: Biological Process
MF: Molecular Function
CC: Cellular Component
