# Supplementary material for: N-Acetyl-L-cysteine facilitates tendon repair and promotes the tenogenic differentiation of tendon stem/progenitor cells by enhancing the integrin α5/β1/PI3K/AKT signaling
Source: BMC Mol Cell Biol. 2023 Jan 5;24:1. doi: 10.1186/s12860-022-00463-0 (PMC9814204; doi:10.1186/s12860-022-00463-0)
Supplement: Supplementary file 2 — Additional file 2. TableS2. Histological scoring. [file 12860_2022_463_MOESM2_ESM.docx]

**Table S2. Histological scoring**

| **Evaluation indicators** | **Score** |
| --- | --- |
| **Fiber arrangement** | |
| Dense, neatly arranged | 2 |
| Partially dense, loose, or disordered | 1 |
| Cluttered and disordered | 0 |
| **Number of cells** | |
| Normal | 2 |
| Increased local cell density | 1 |
| Abnormal increase of cells and decreased ECM | 0 |
| **Cell arrangement** | |
| Uniaxial arrangement | 2 |
| 10–50% of cells are irregularly arranged | 1 |
| More than 50% cells are irregularly arranged | 0 |
| **Cell distribution** | |
| Uniform and physiological | 1 |
| Cell clustering | 0 |
| **Nuclear morphology** | |
| Elongated or heterochromatic nucleus | 2 |
| Large, oval, polymorphic heterochromatin nuclei in 10–30% of nuclei | 1 |
| Large, oval, euchromatin and heterochromatin nuclei in more than 30% of nuclei | 0 |
| **Components of the scar in the repaired areas** | |
| Homogeneous (one component) | 2 |
| Local abnormal tissue composition | 1 |
| The entire tissue is completely replaced by other ingredients | 0 |
| **Appearance** | |
| Normal form, normal gloss | 2 |
| Partial thickening and reduced gloss | 1 |
| Significantly thicker, less glossy, less elastic and less rigid | 0 |
| **Tissue metaplasia** | |
| None | 3 |
| Partial swelling | 2 |
| Fatty infiltration or scar fibrosis | 1 |
| Cartilage or heterotopic ossification | 0 |
| **Neovascularization** | |
| Few new capillaries | 1 |
| Significant increase in new blood vessels | 0 |
| **Inflammation** | |
| No inflammatory cells | 1 |
| Inflammatory cell infiltration | 0 |
