# Supplementary material for: N-Acetyl-L-cysteine facilitates tendon repair and promotes the tenogenic differentiation of tendon stem/progenitor cells by enhancing the integrin α5/β1/PI3K/AKT signaling
Source: BMC Mol Cell Biol. 2023 Jan 5;24:1. doi: 10.1186/s12860-022-00463-0 (PMC9814204; doi:10.1186/s12860-022-00463-0)

Fig2B SCX

Three times repeated exposure image

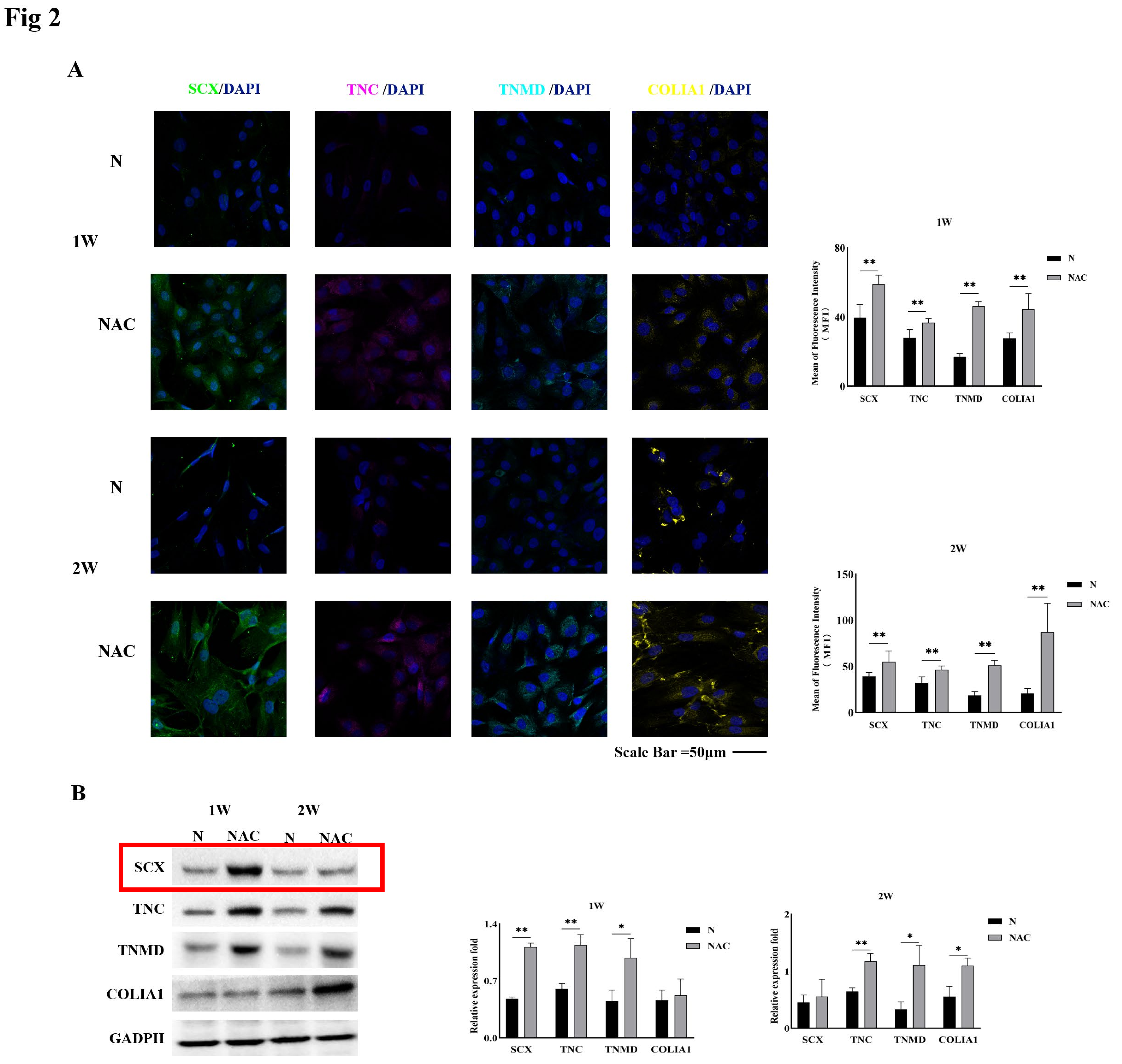

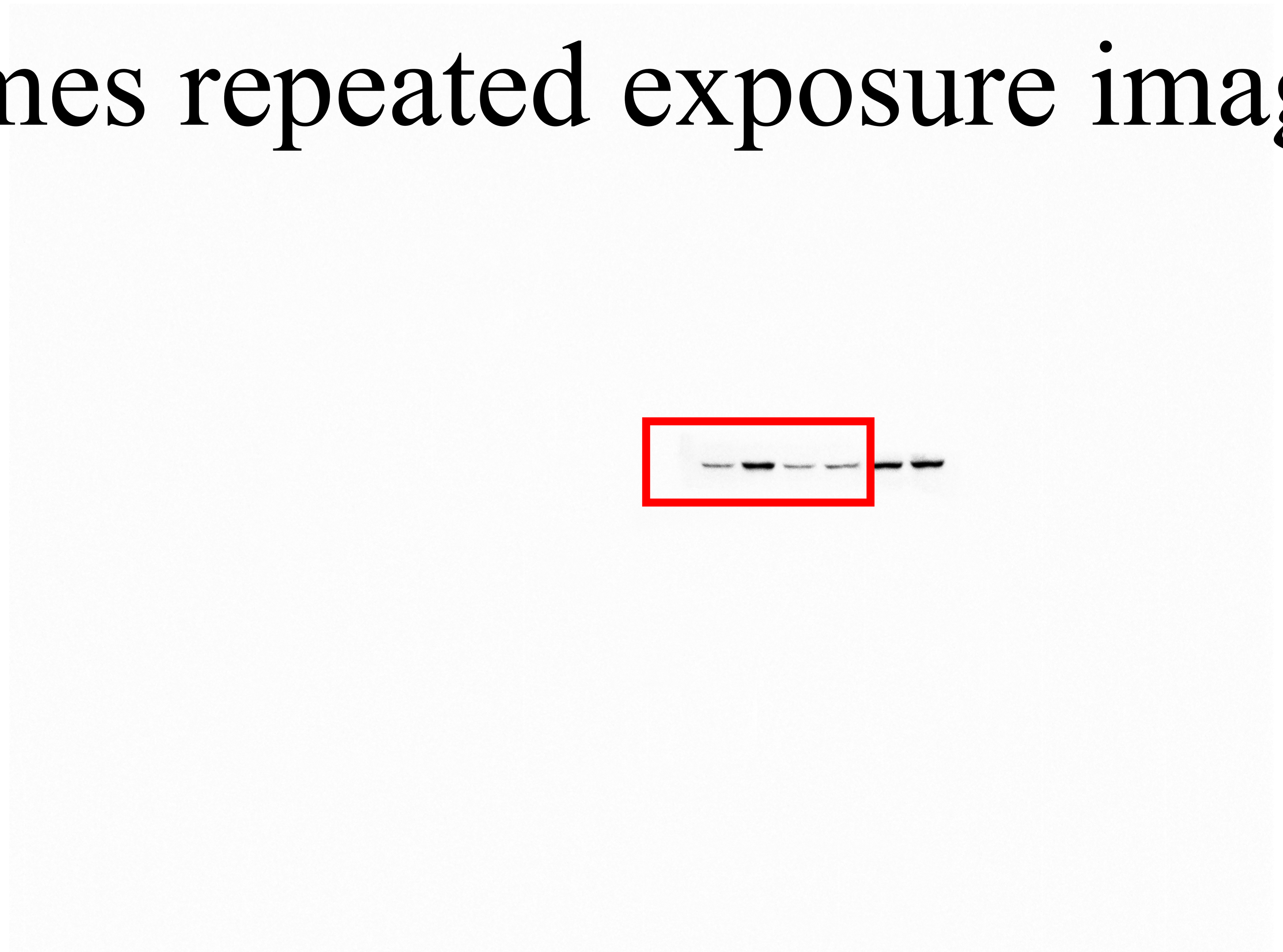

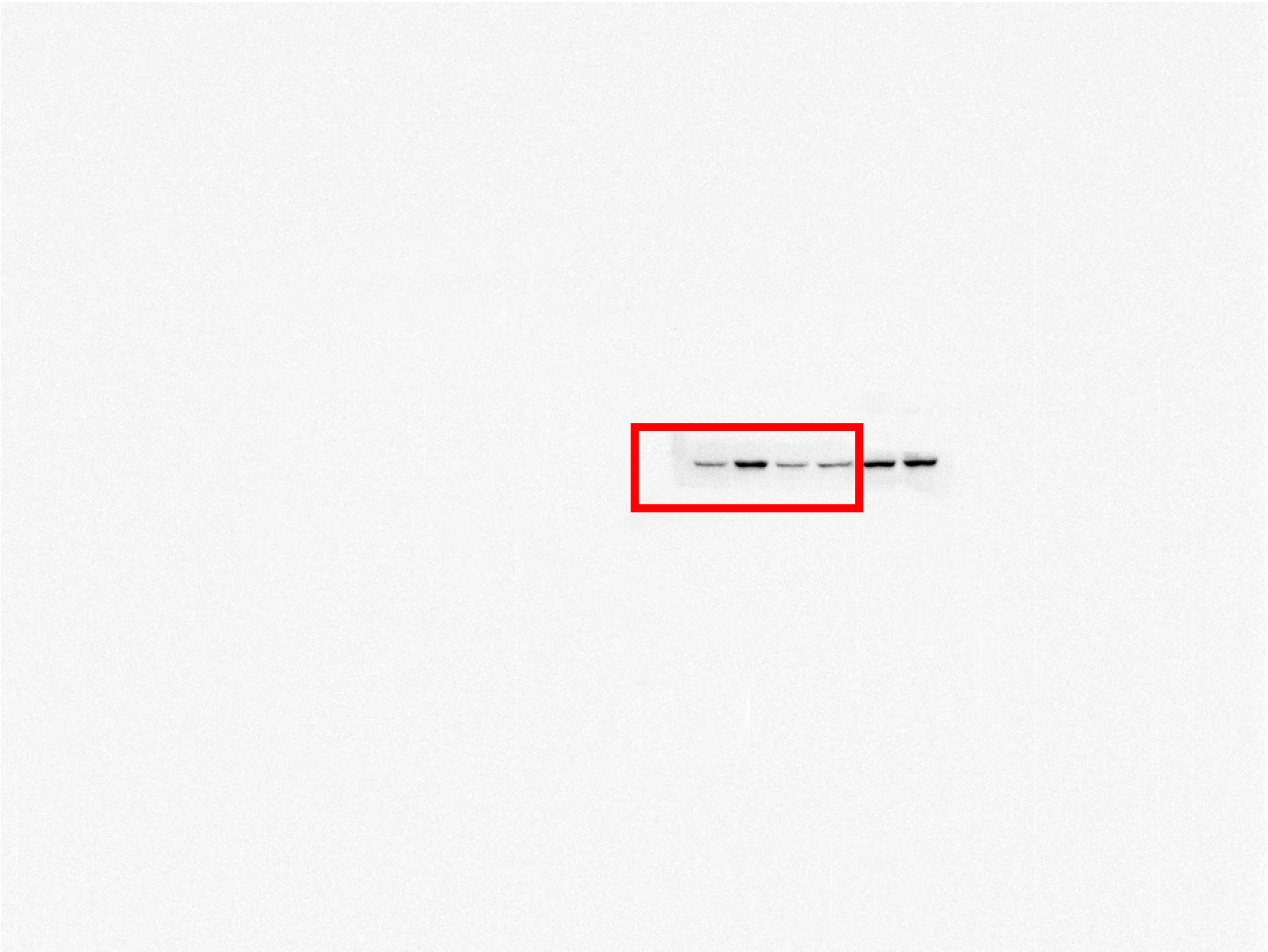

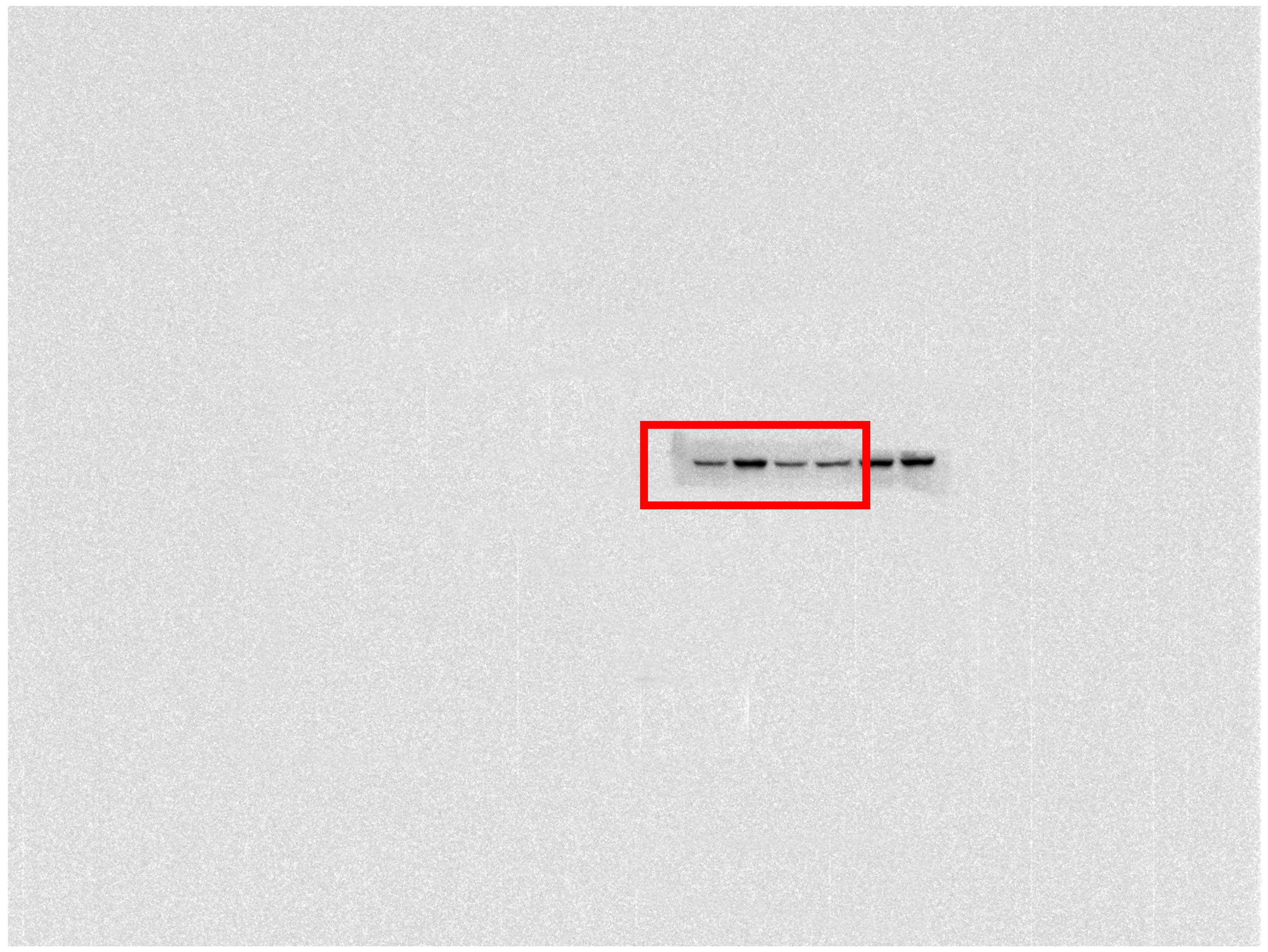

Fig2B TNC

Three times repeated exposure image

Fig 2

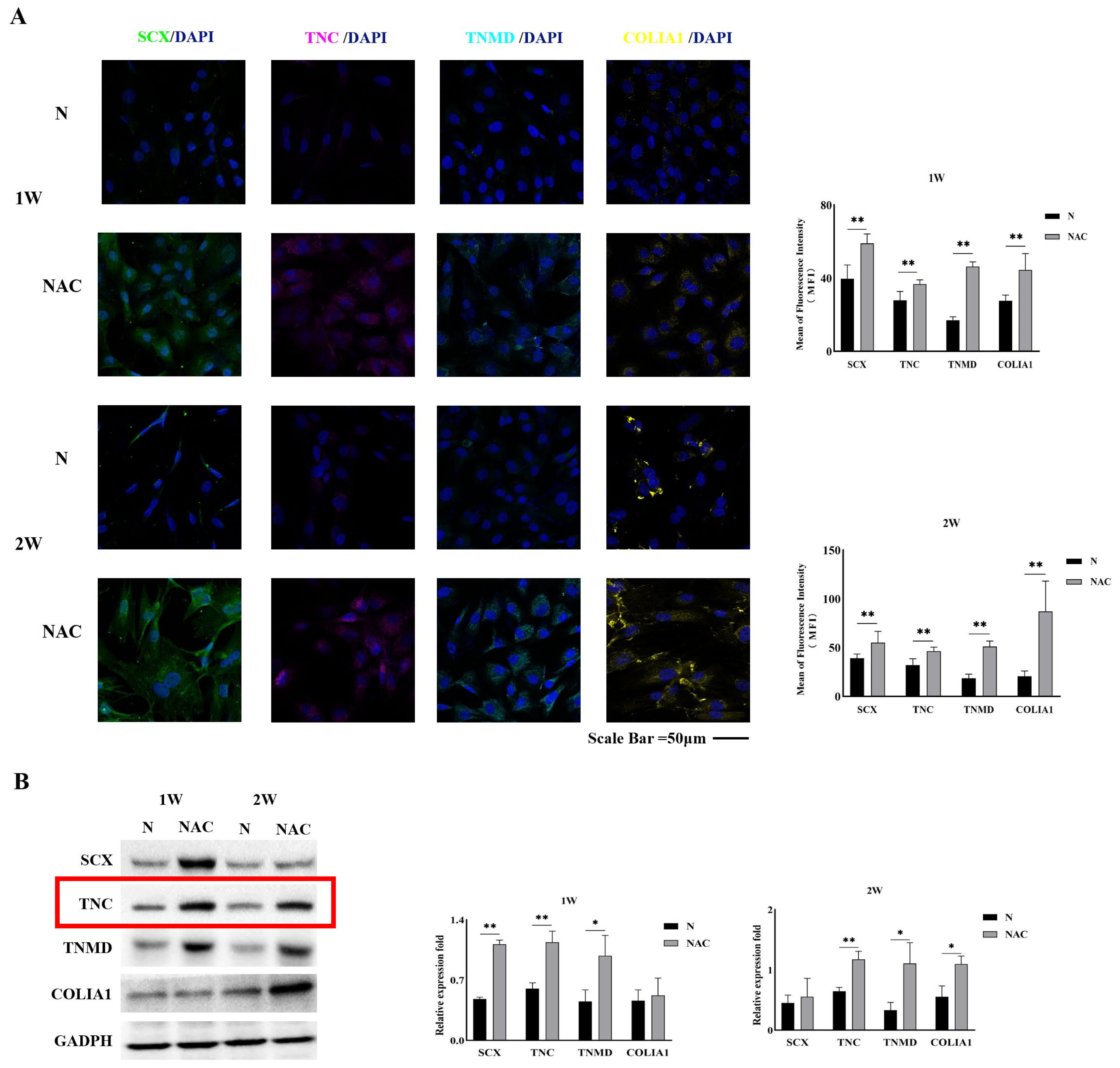

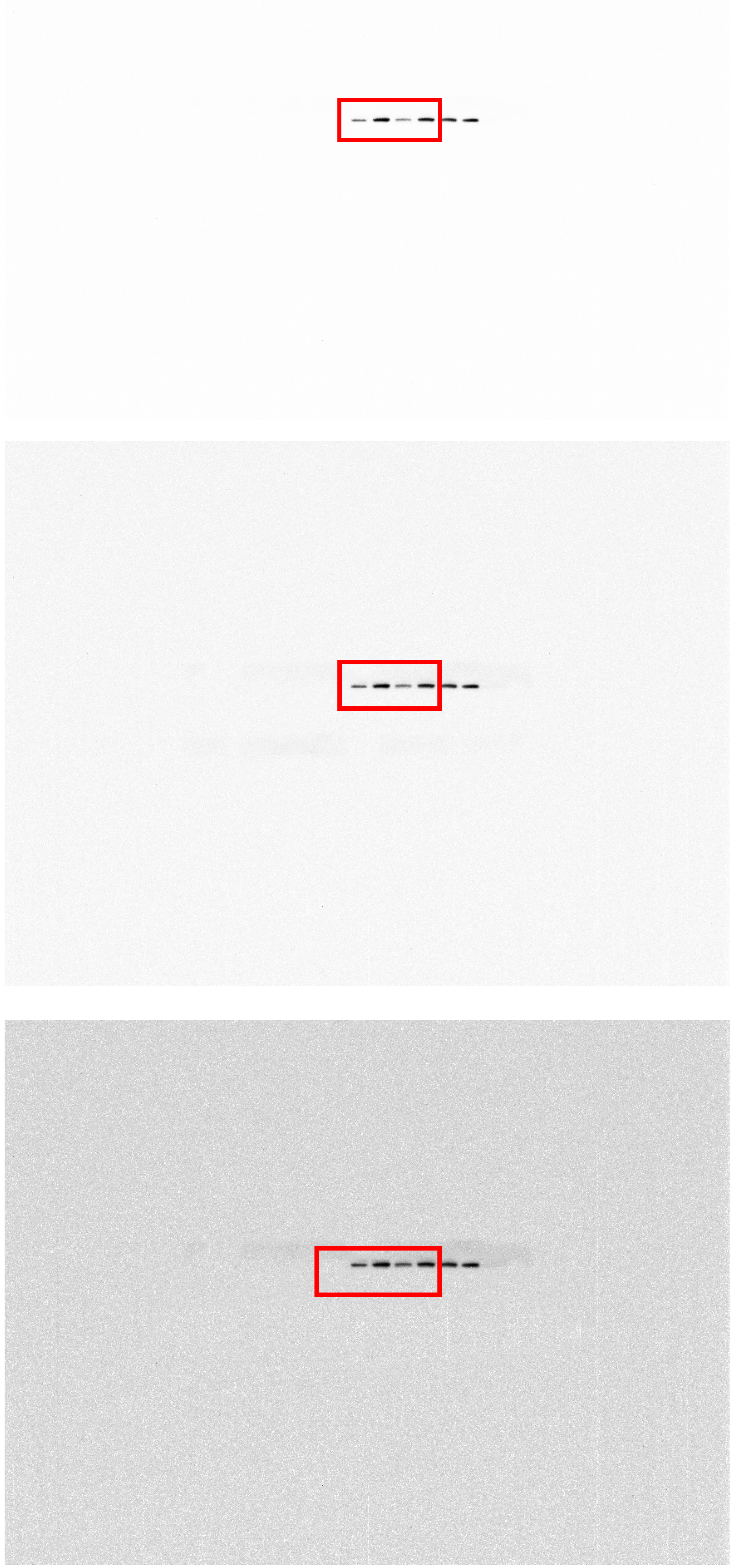

Fig2B TNMD

Three times repeated exposure image

Fig 2

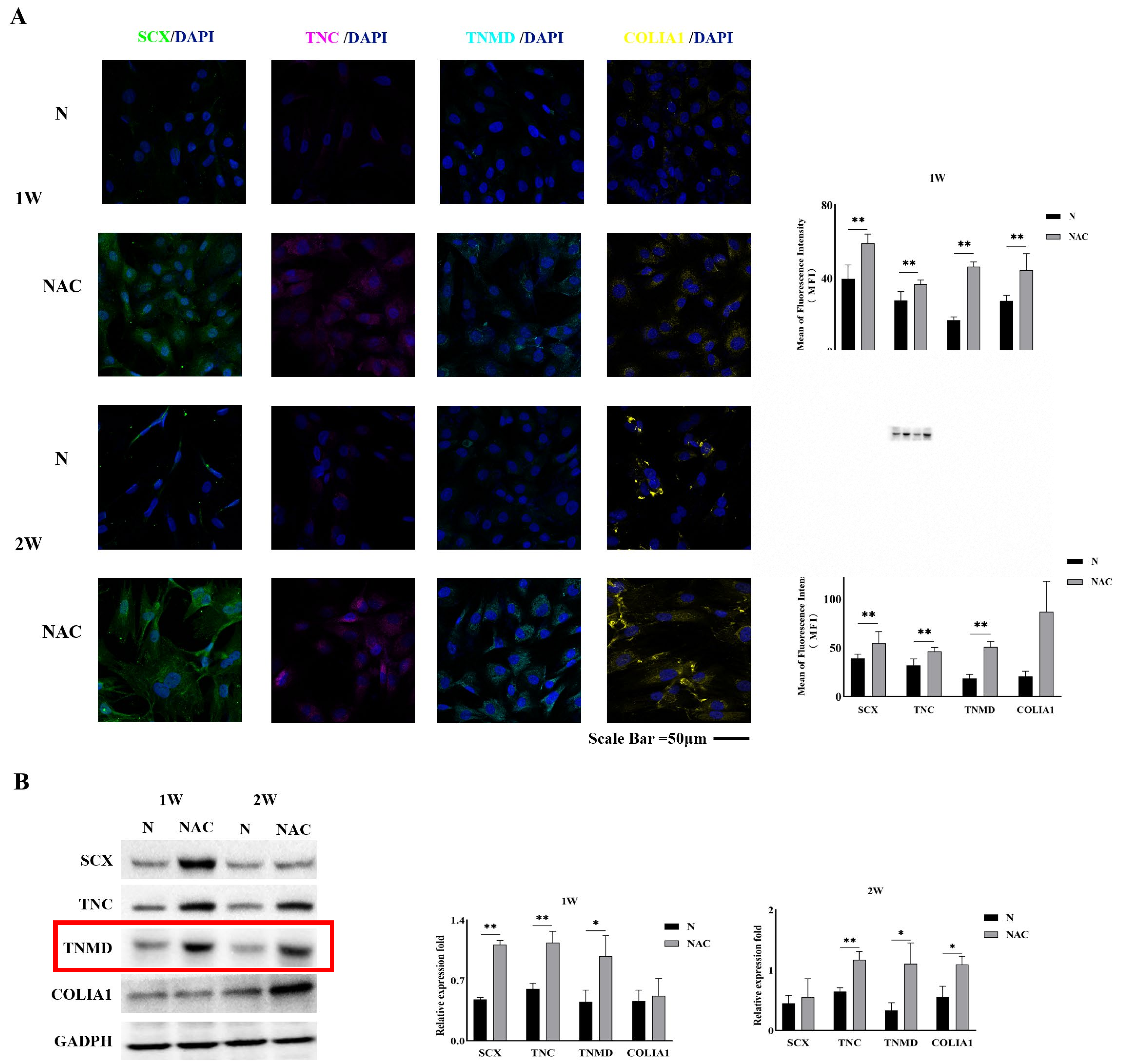

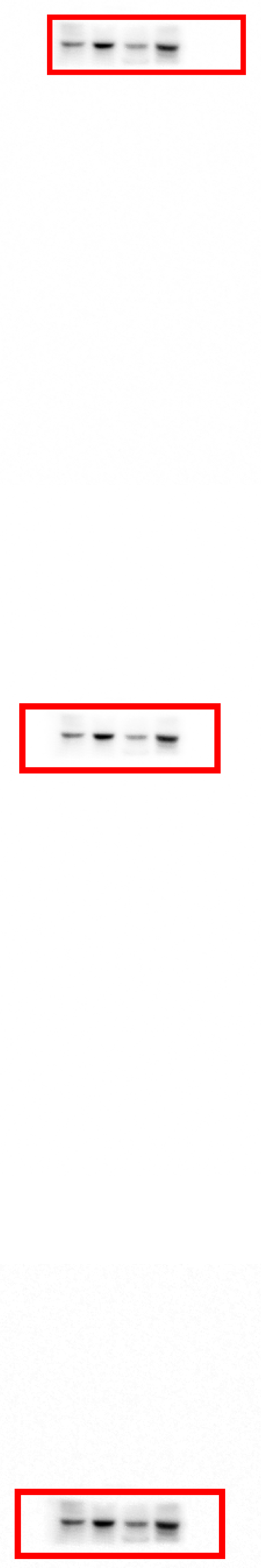

Fig2B COLIA1

Three times repeated exposure image

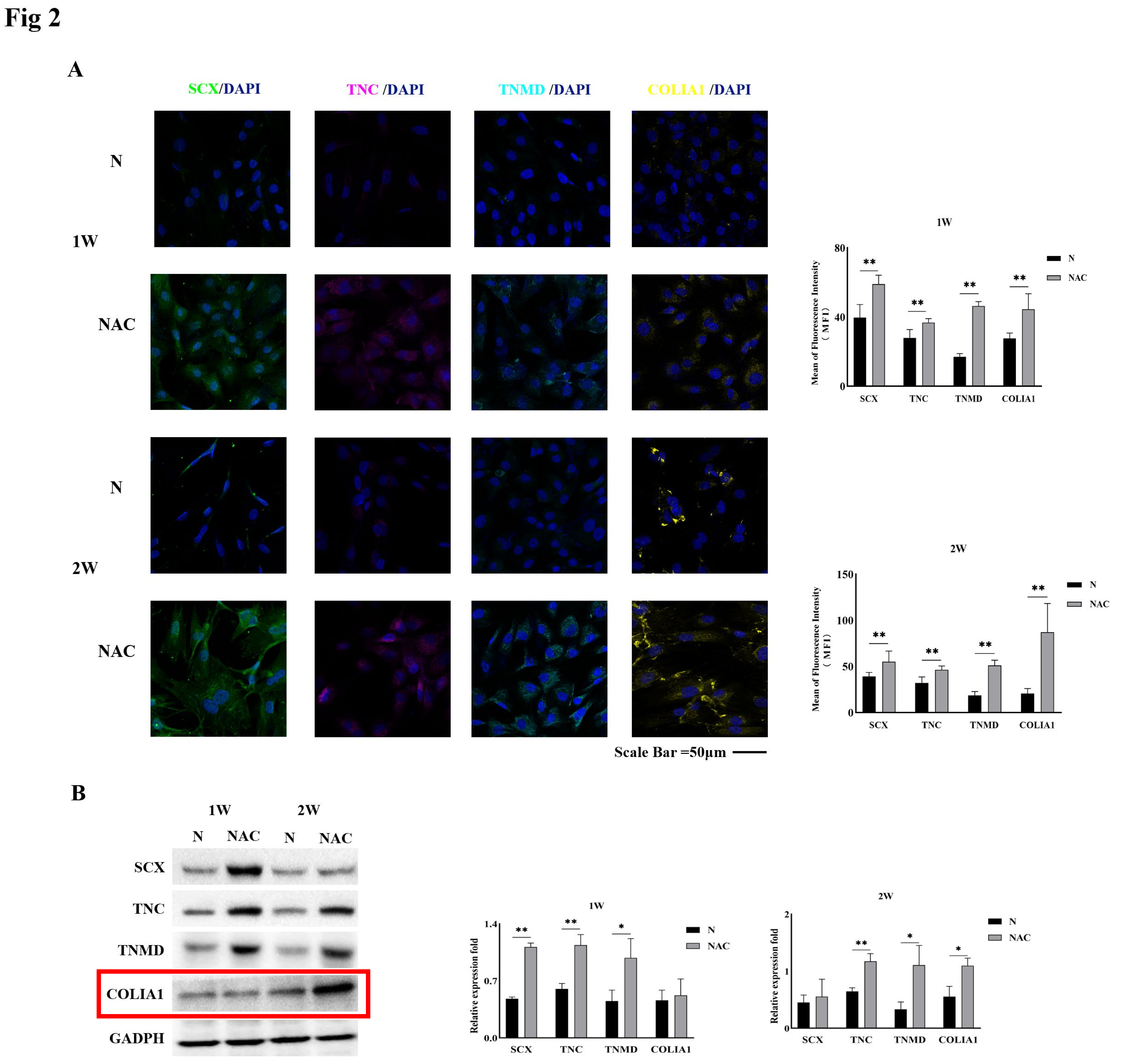

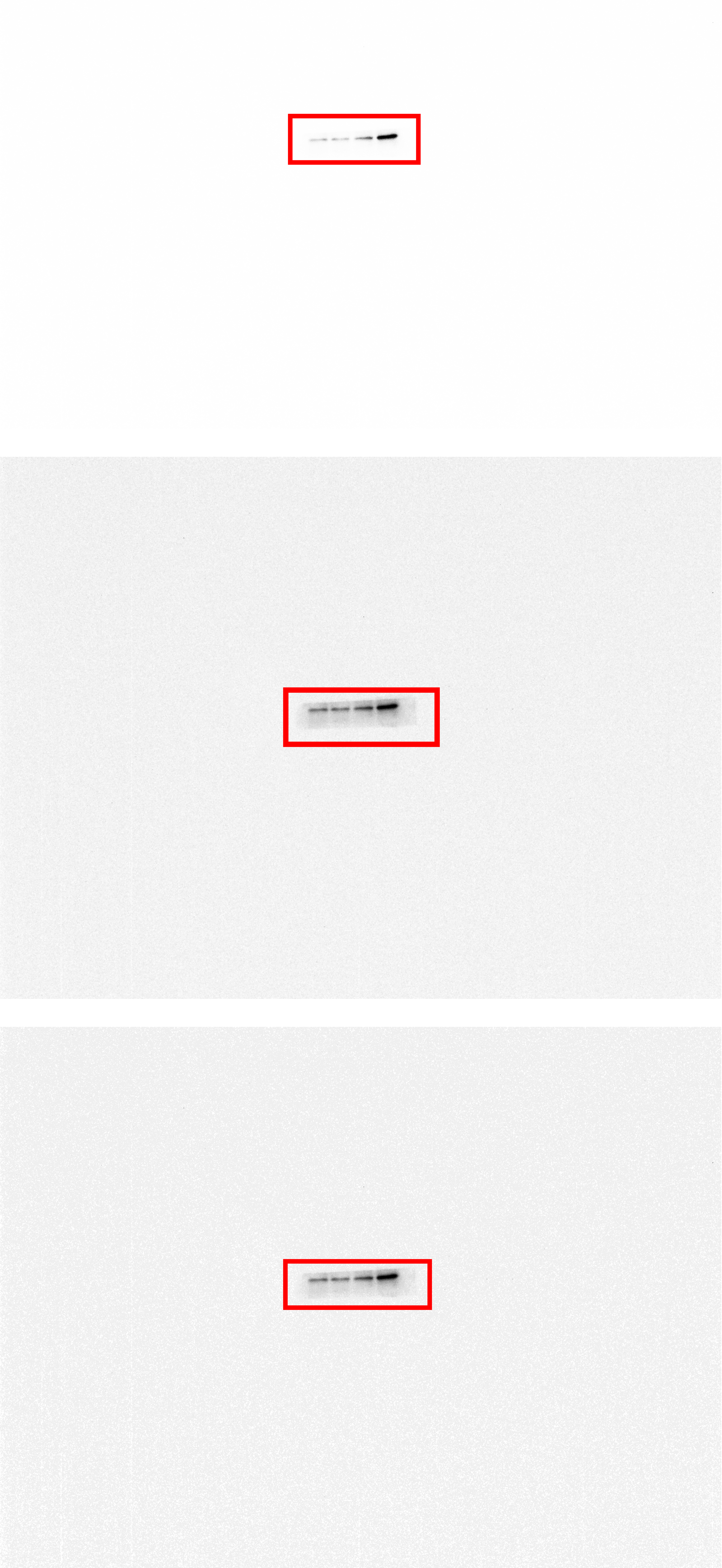

Fig2B GADPH

Three times repeated exposure image

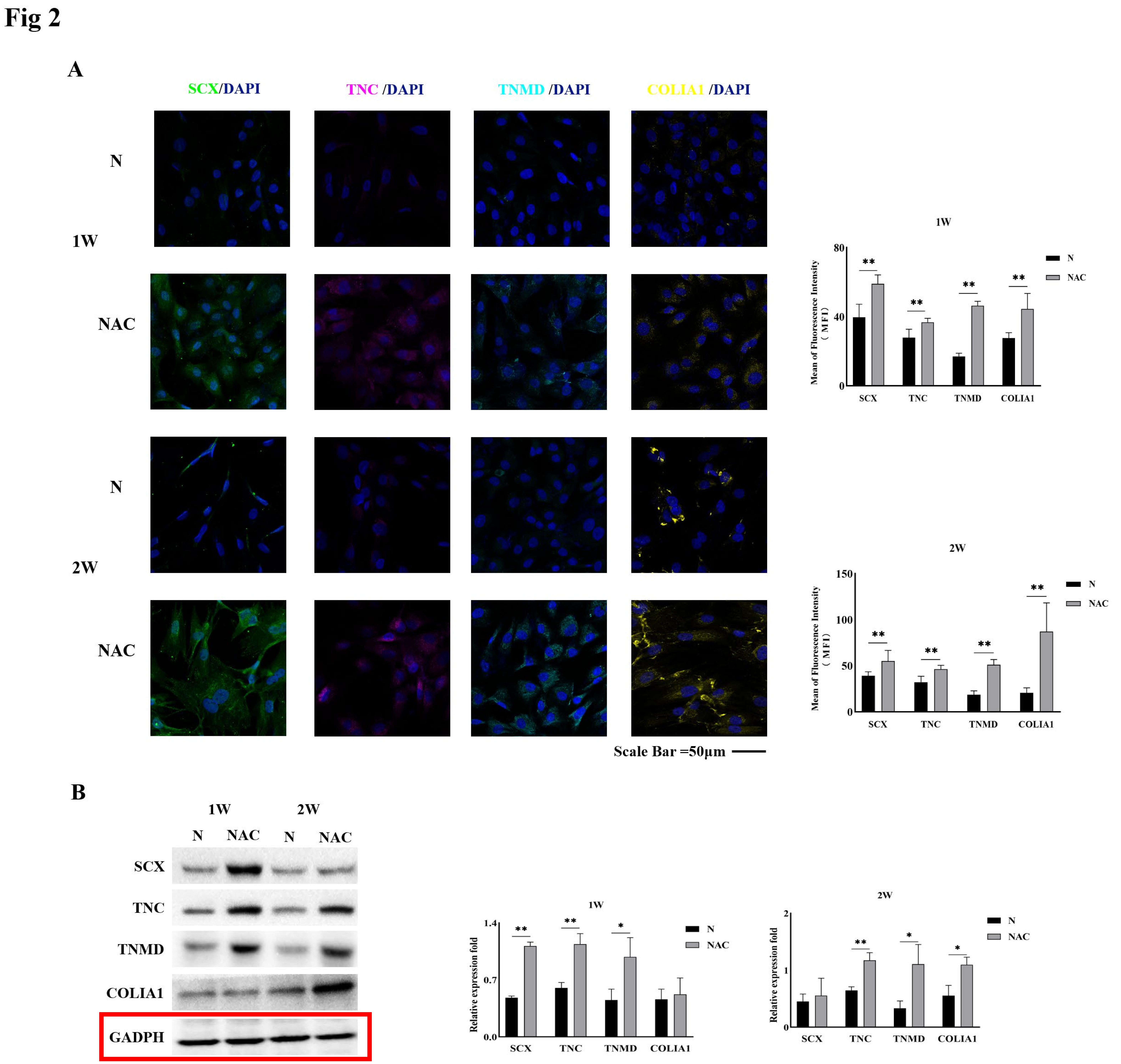

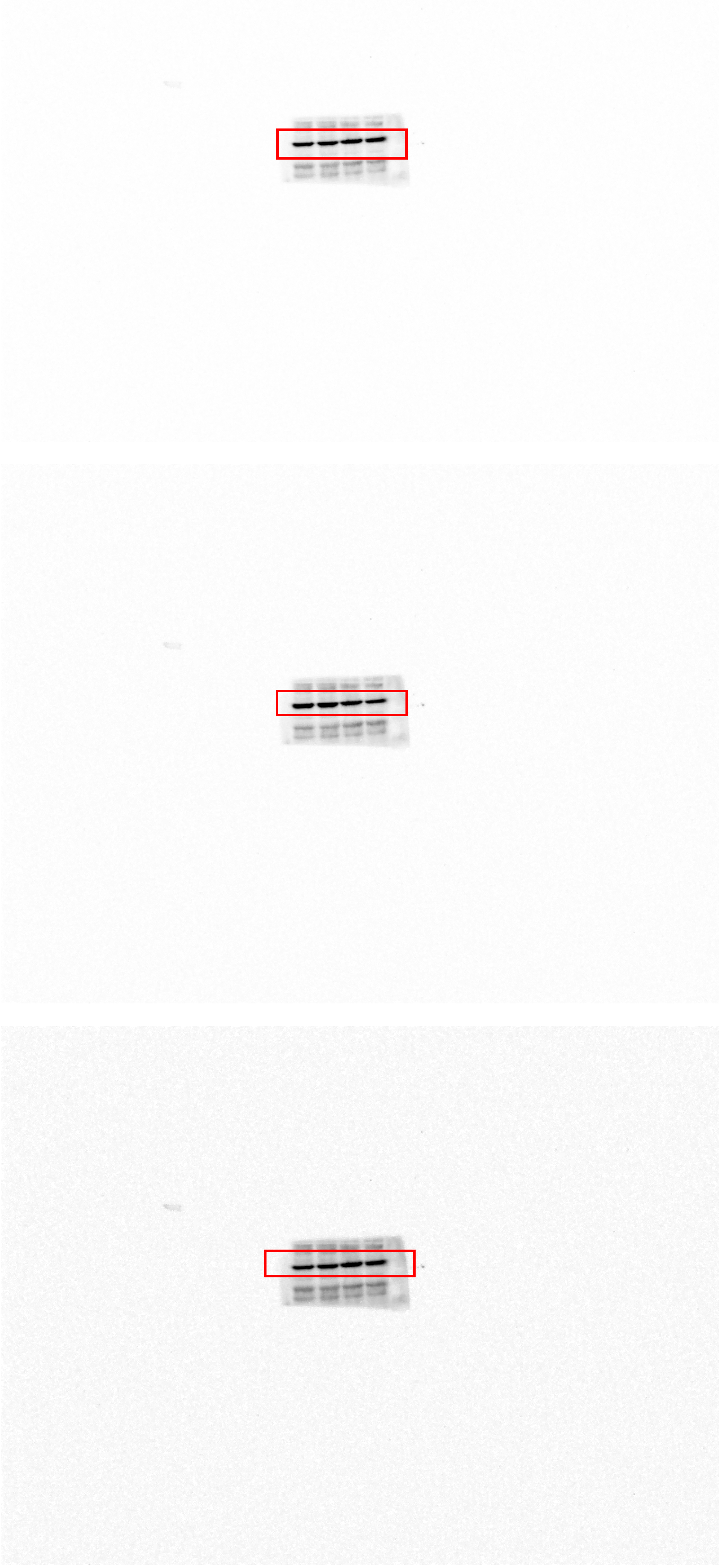

Supplement: Supplementary file 3 — Additional file 3. [file 12860_2022_463_MOESM3_ESM.pdf]
