# Supplementary material for: N-Acetyl-L-cysteine facilitates tendon repair and promotes the tenogenic differentiation of tendon stem/progenitor cells by enhancing the integrin α5/β1/PI3K/AKT signaling
Source: BMC Mol Cell Biol. 2023 Jan 5;24:1. doi: 10.1186/s12860-022-00463-0 (PMC9814204; doi:10.1186/s12860-022-00463-0)

Fig4B PI3K

Three times repeated exposure image

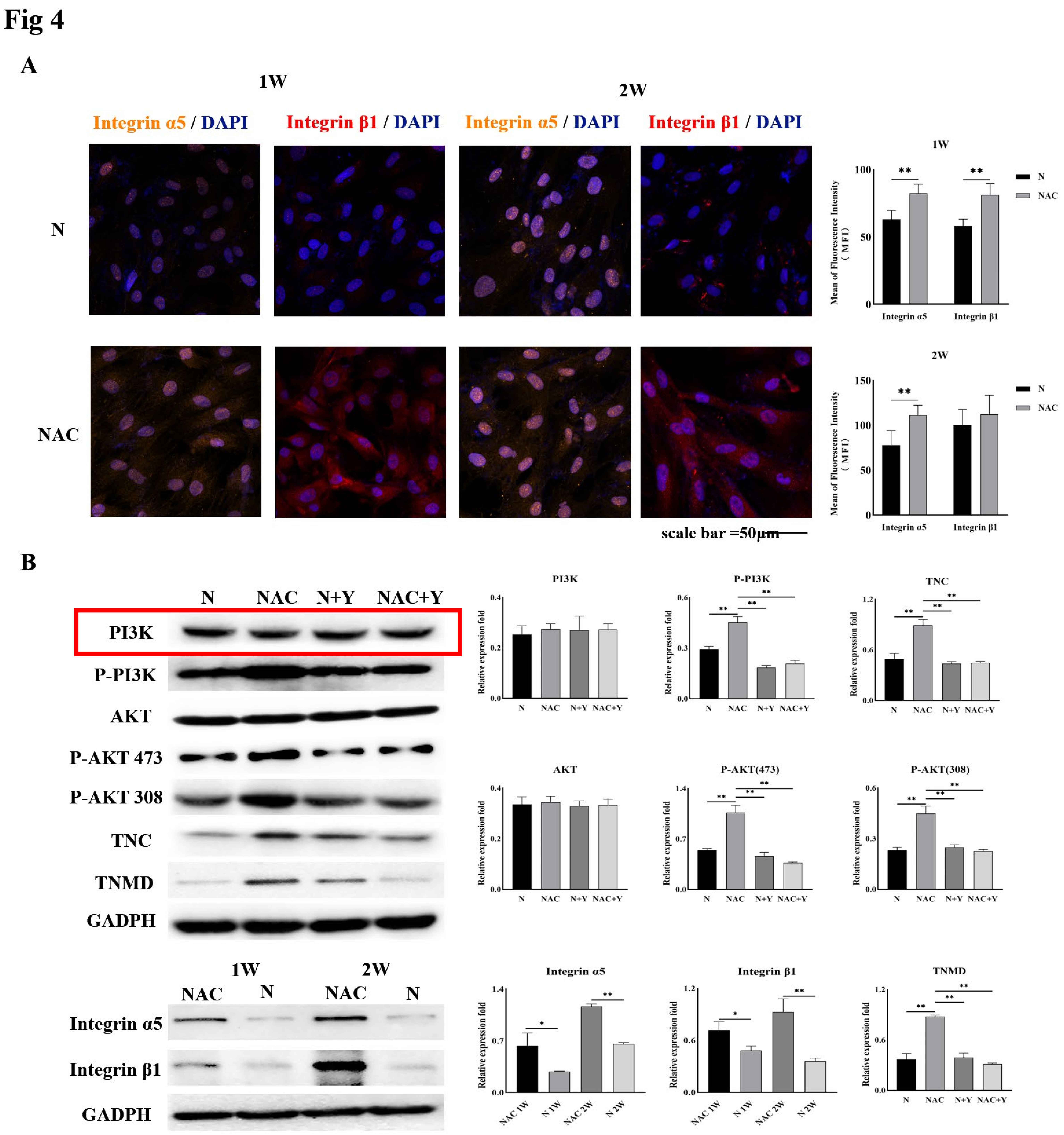

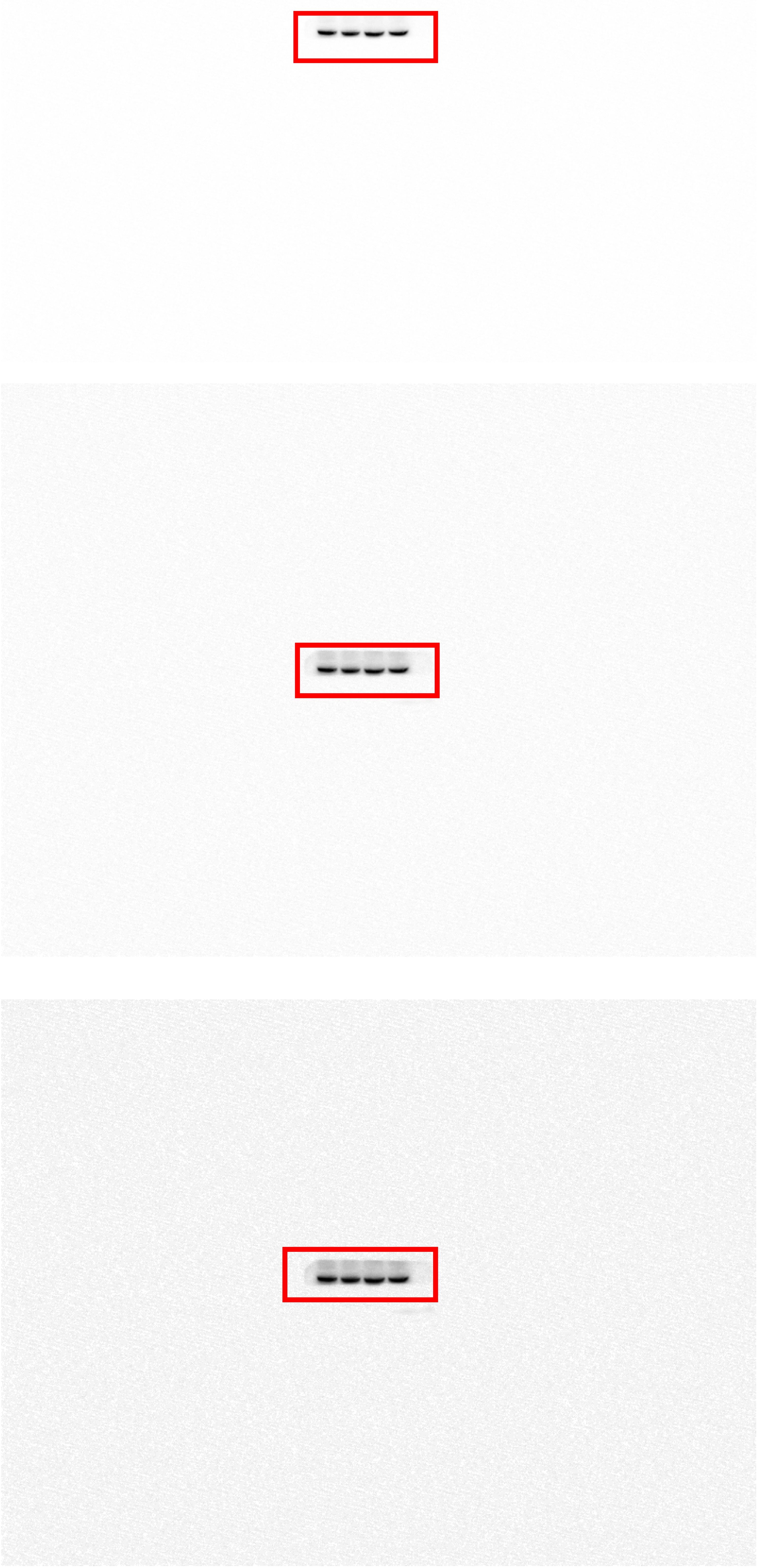

Fig4B P-PI3K

Three times repeated exposure image

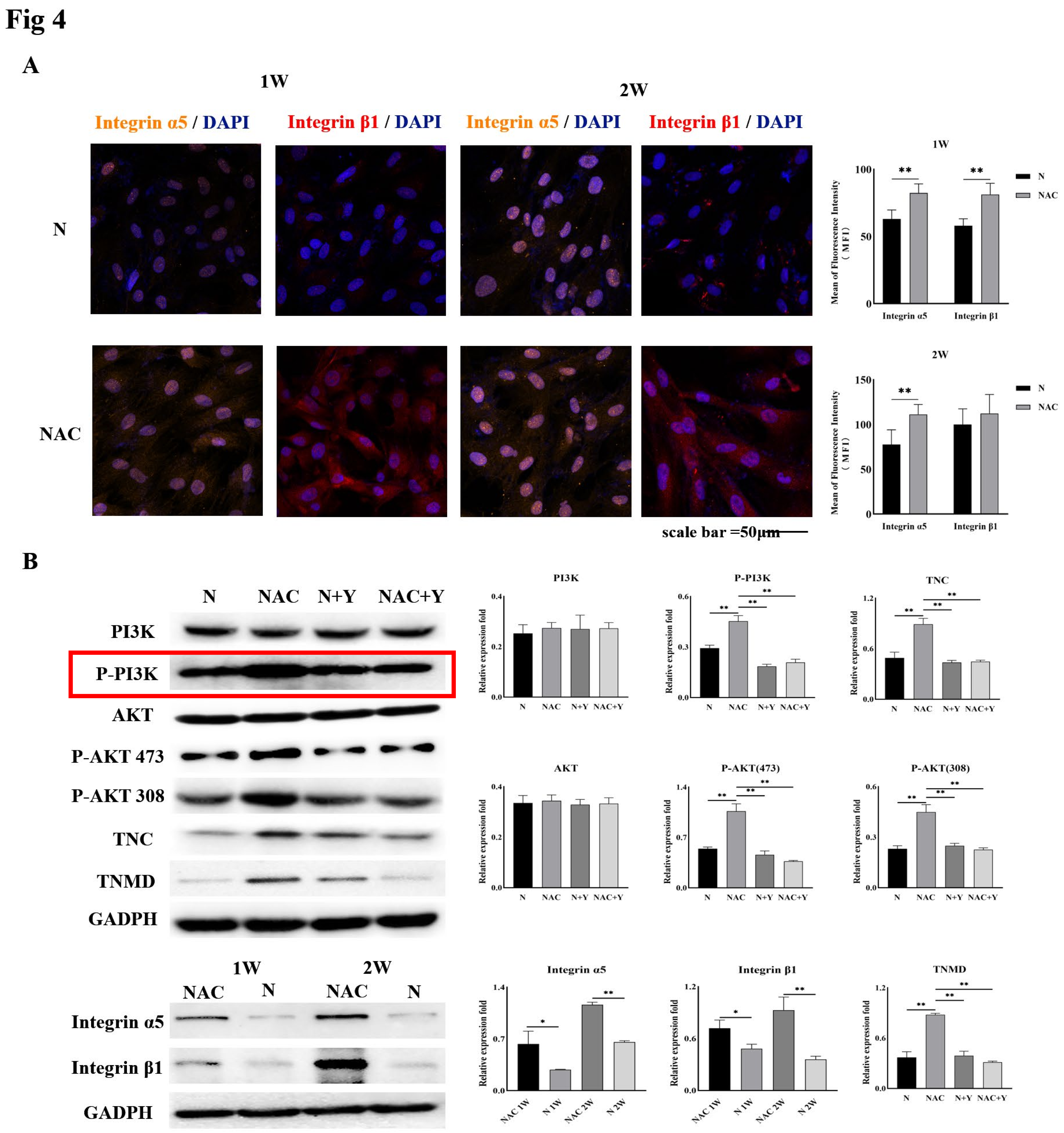

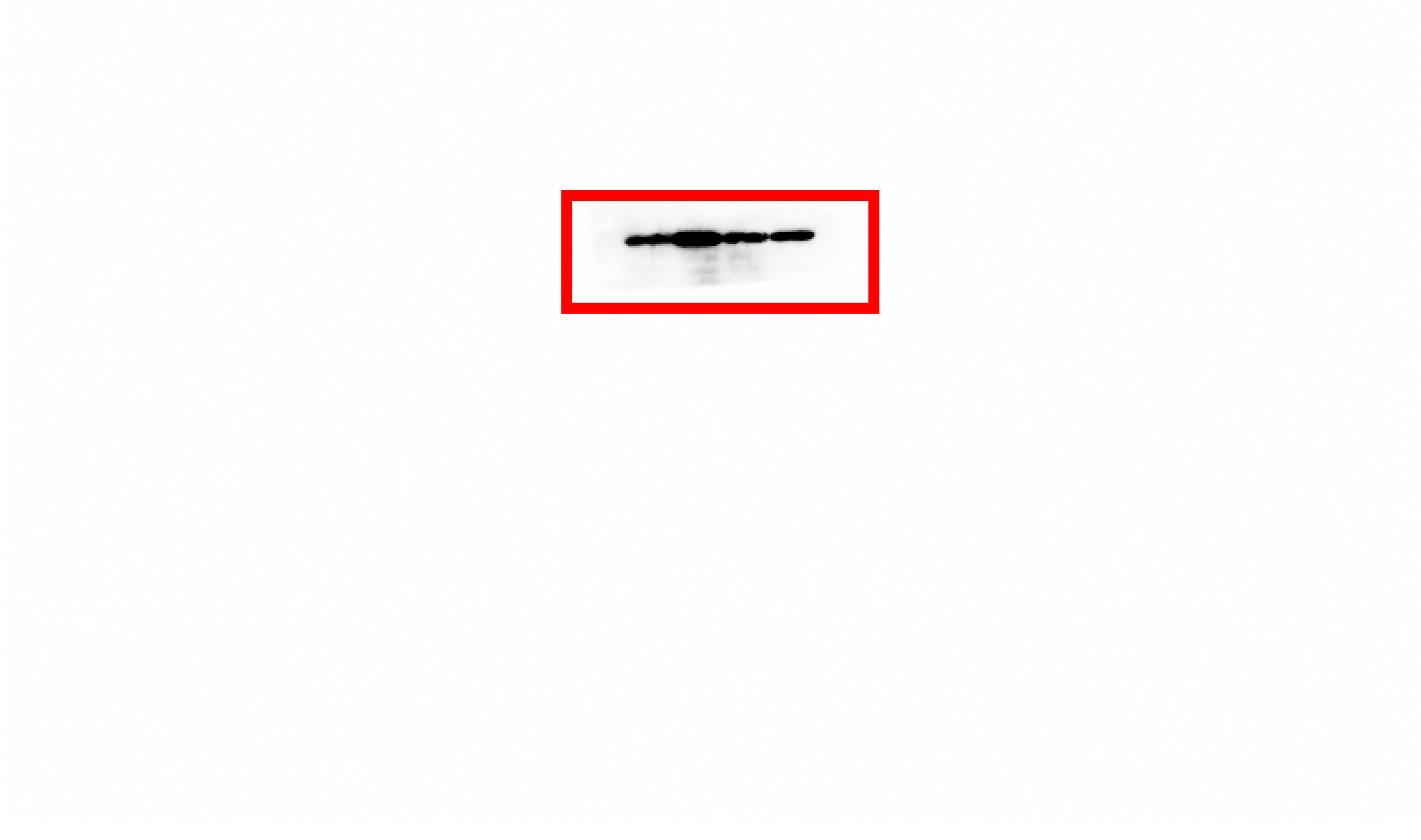

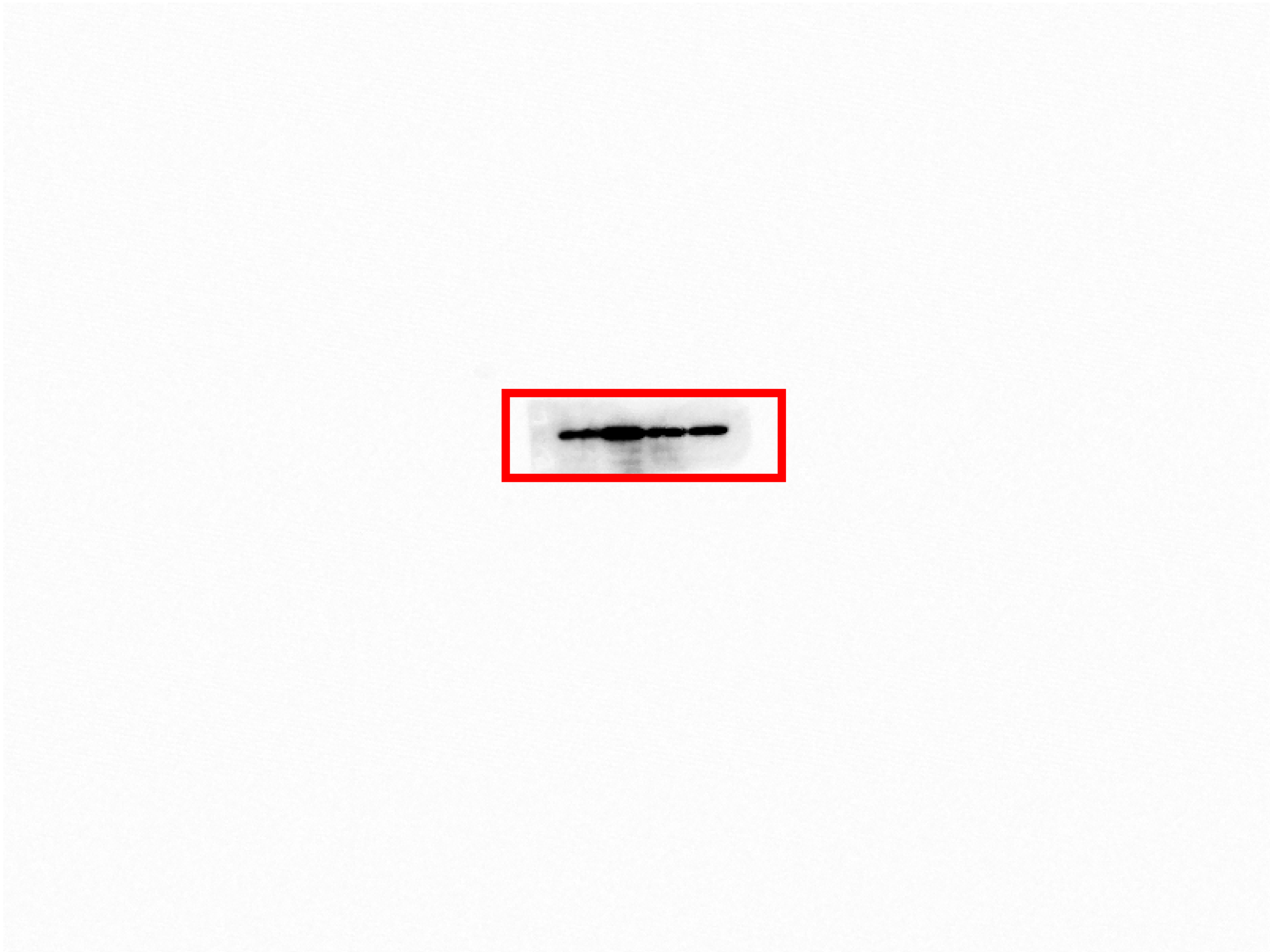

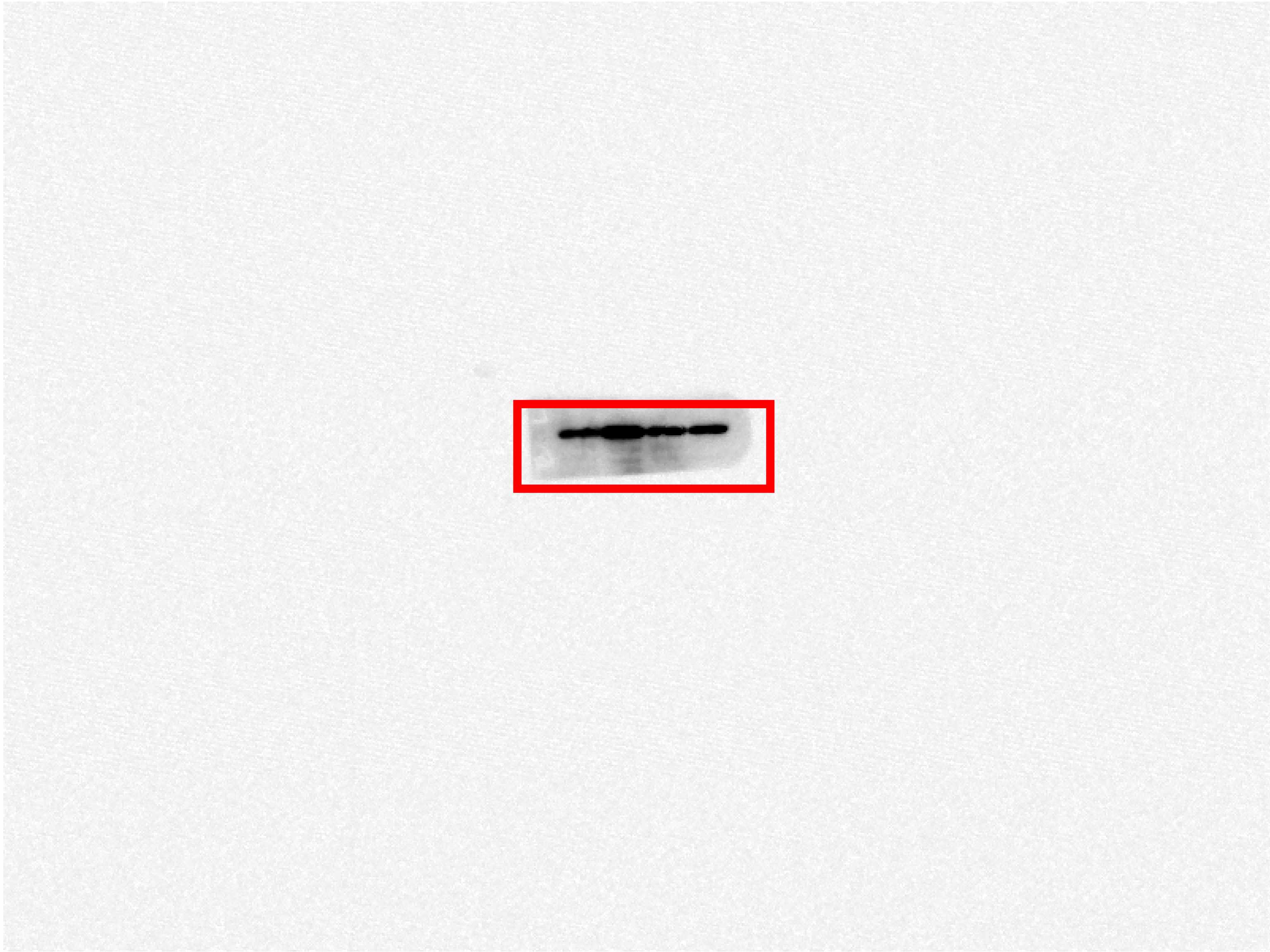

Fig4B AKT

Three times repeated exposure image

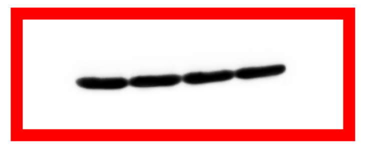

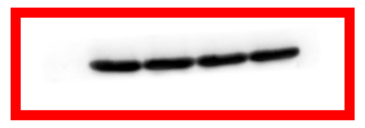

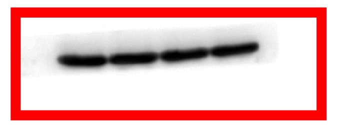

Fig 4

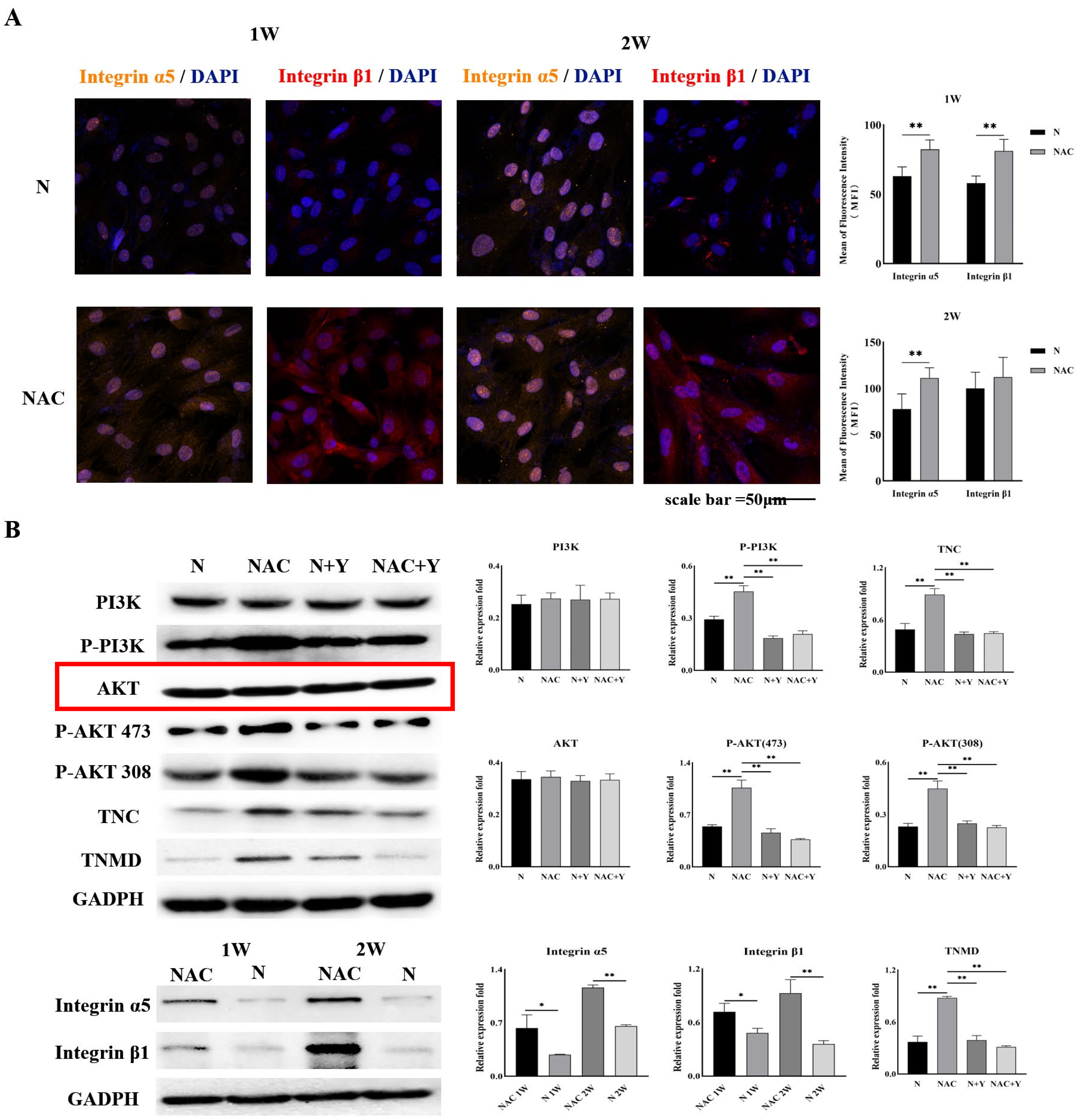

Fig4B P-AKT473

Three times repeated exposure image

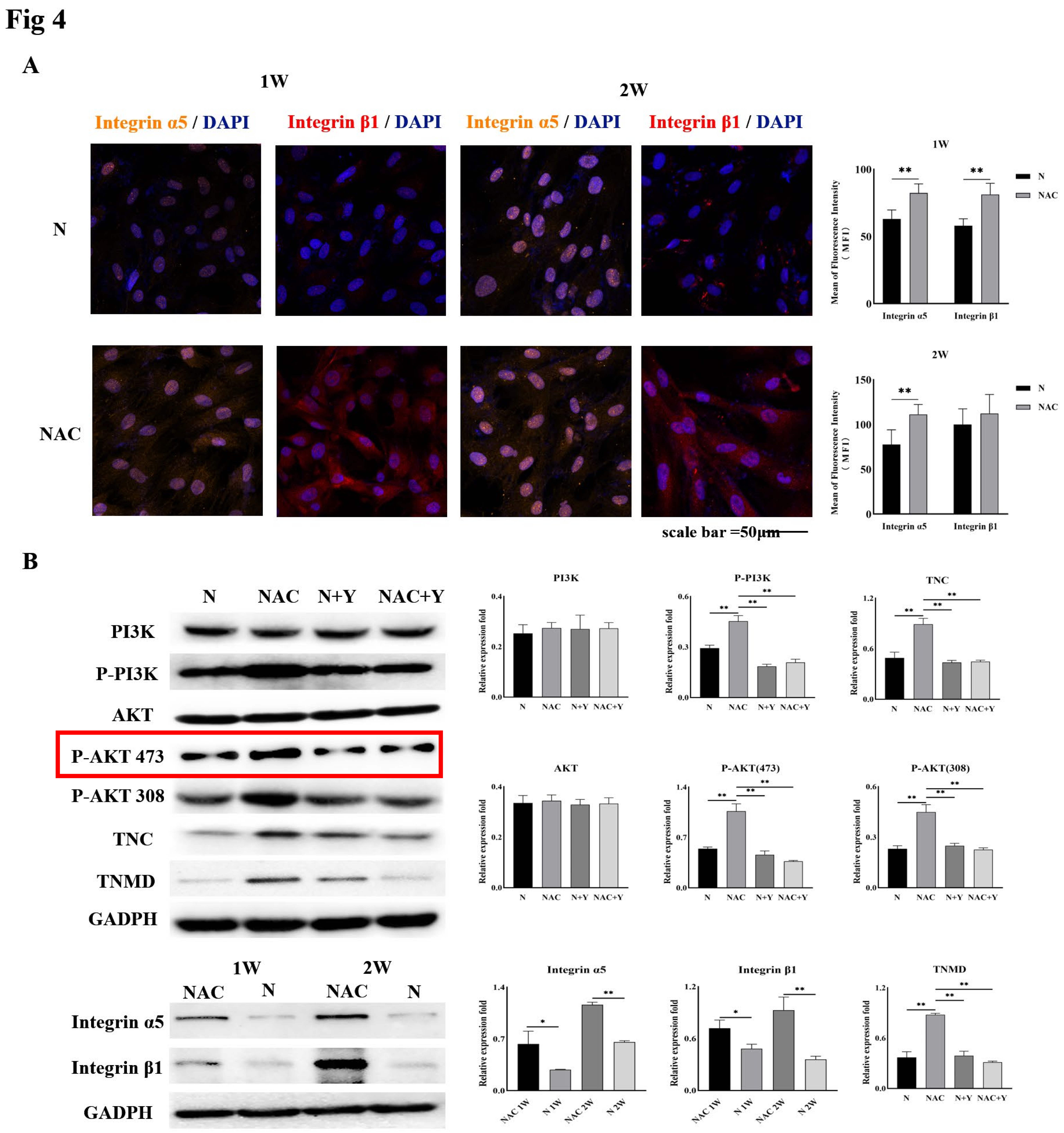

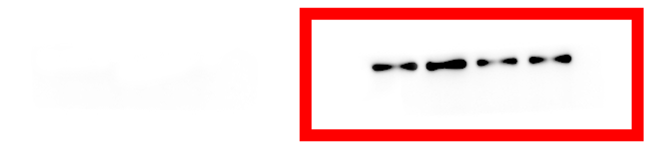

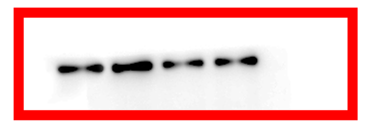

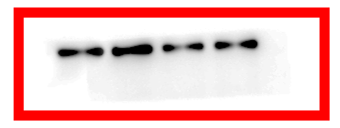

Fig4B P-AKT308

Three times repeated exposure image

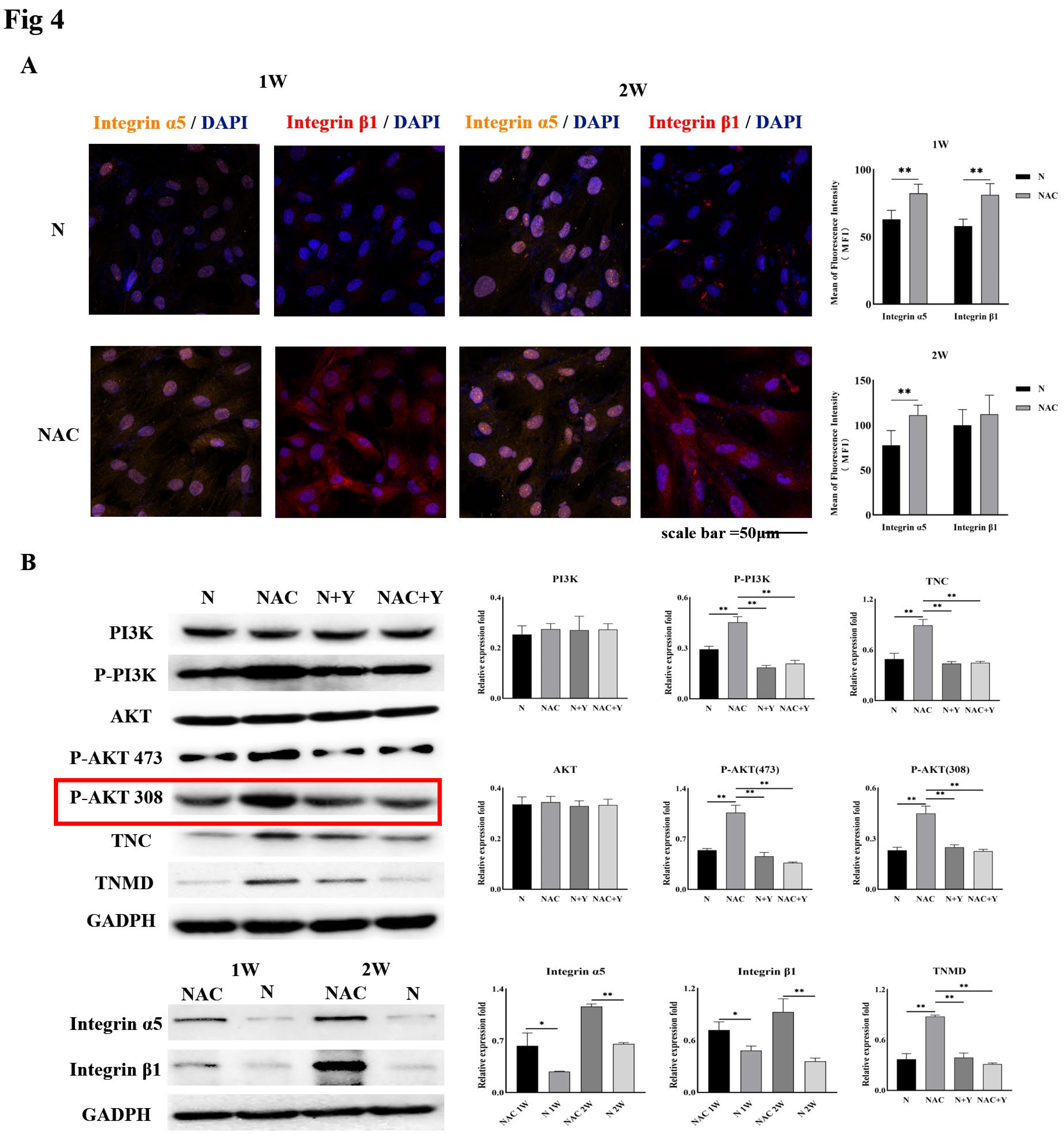

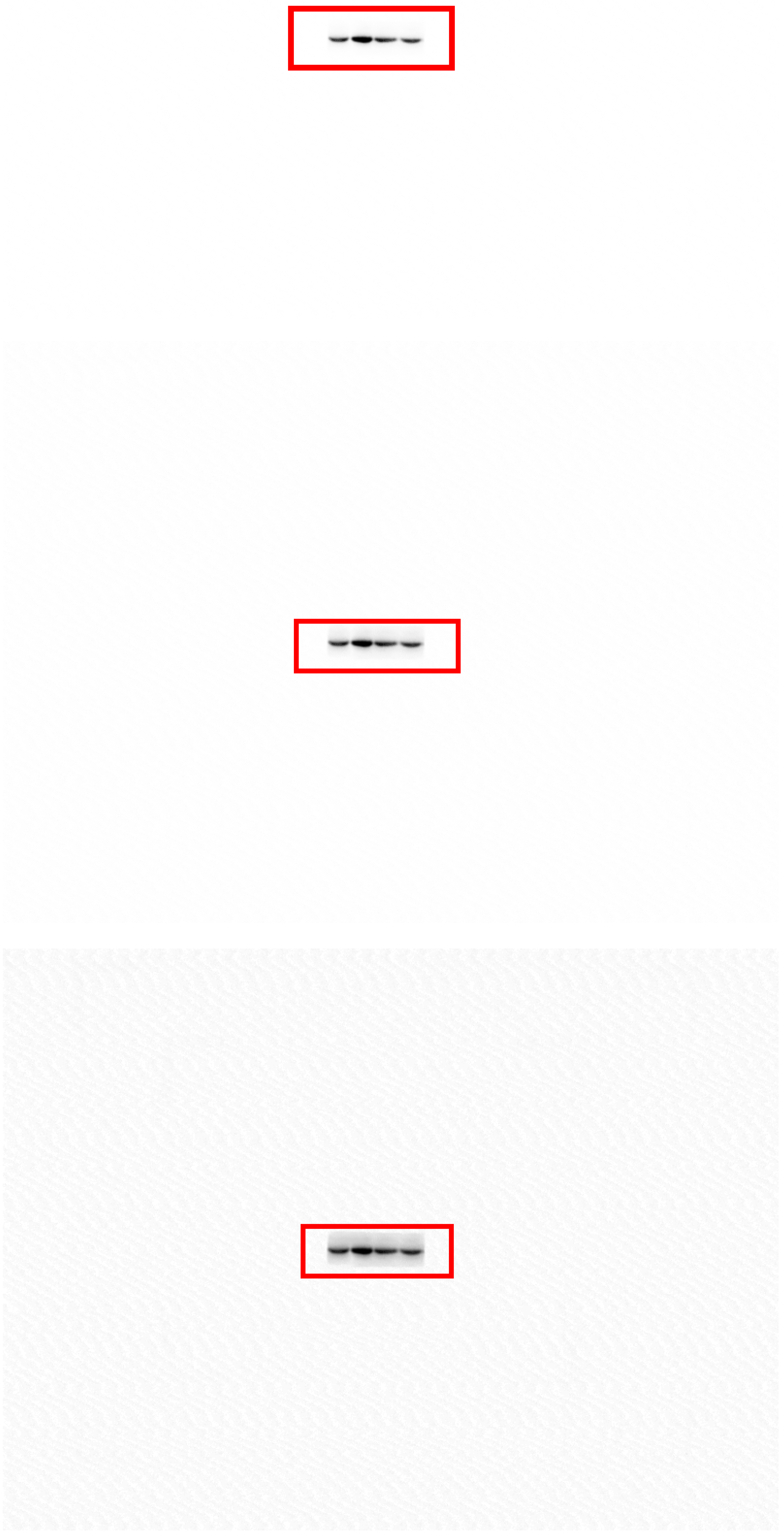

Fig4B TNC

Three times repeated exposure image

Fig 4

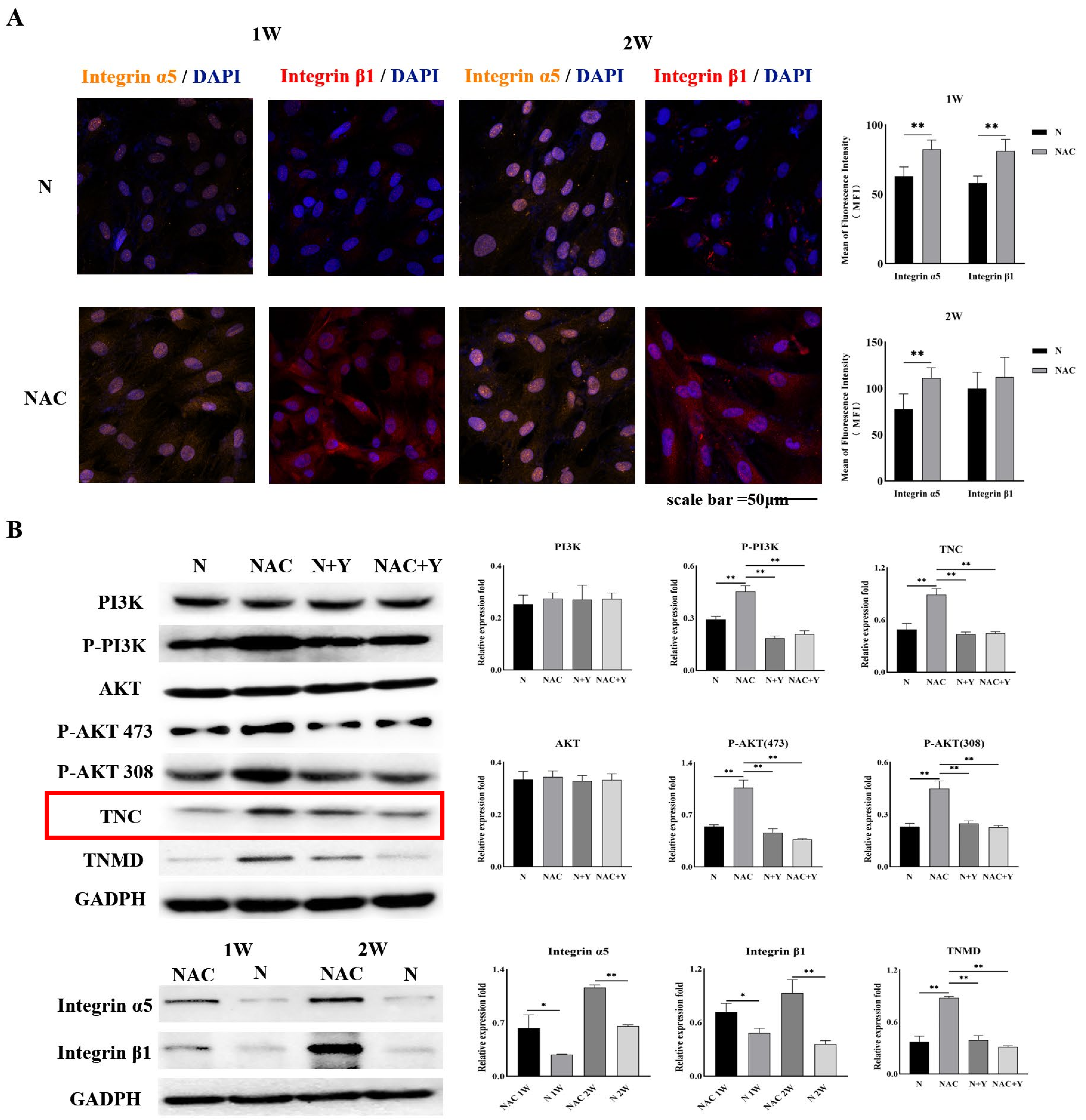

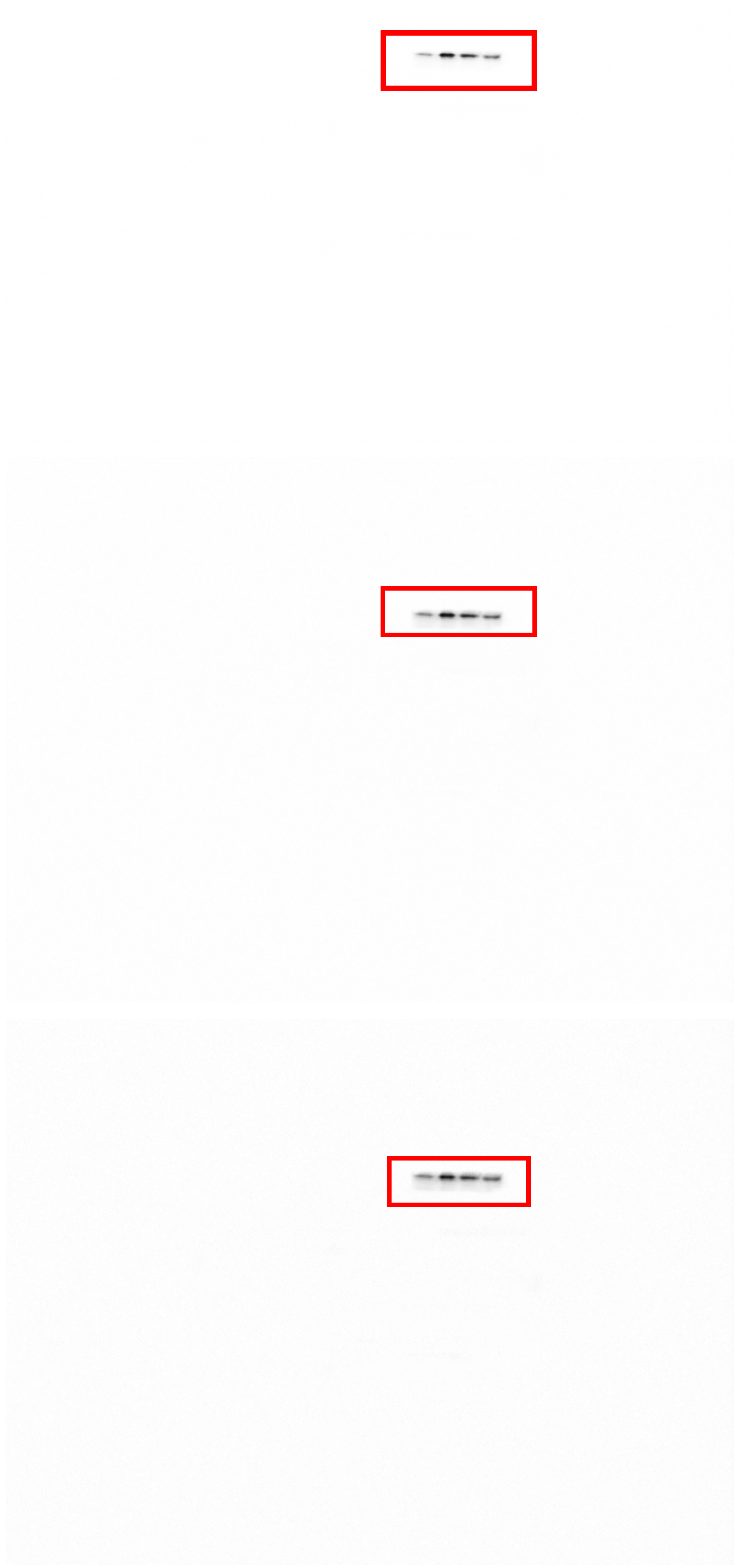

Fig4B TNMD

Three times repeated exposure image

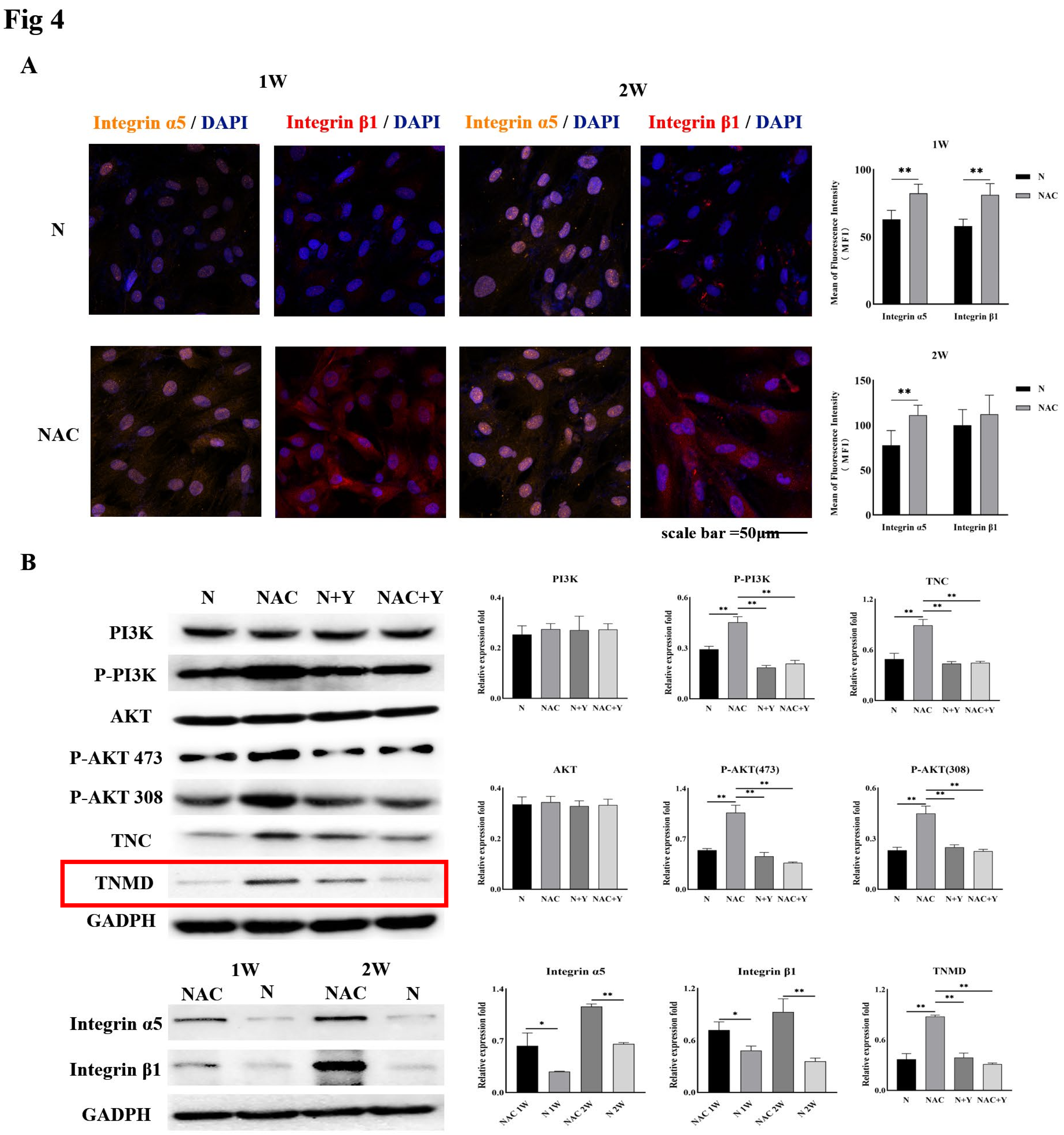

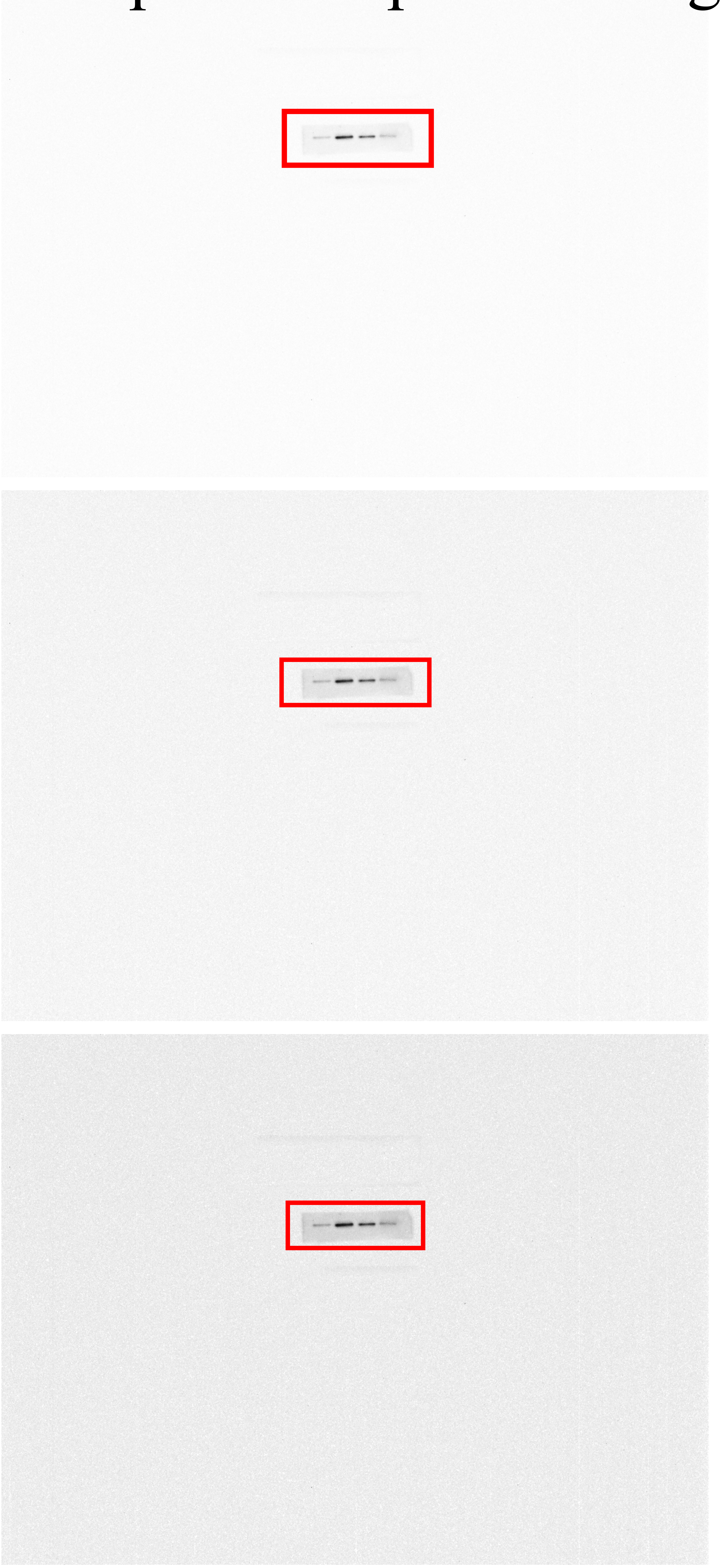

Fig4B PI3K-AKT-GADPH

Three times repeated exposure image

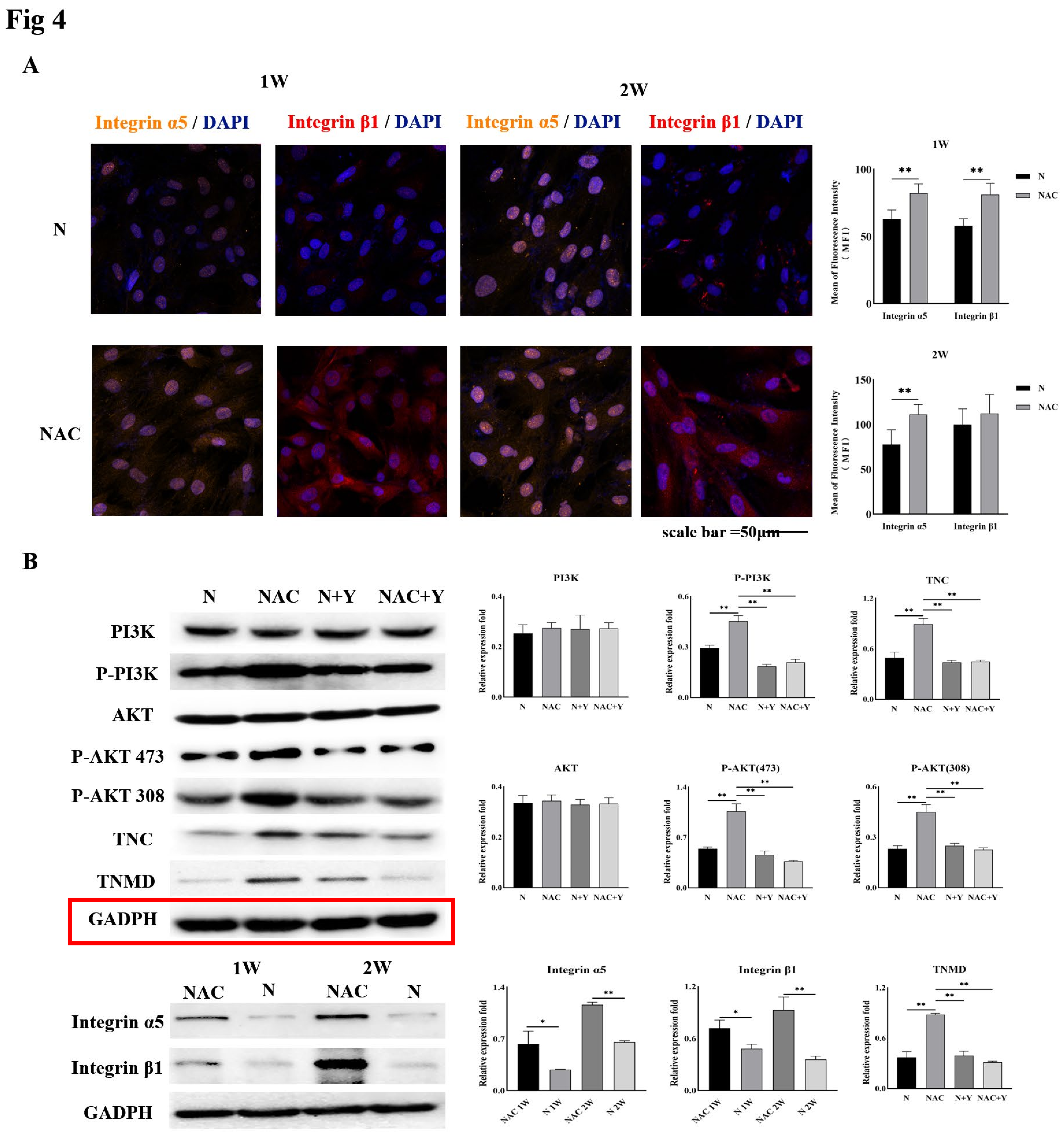

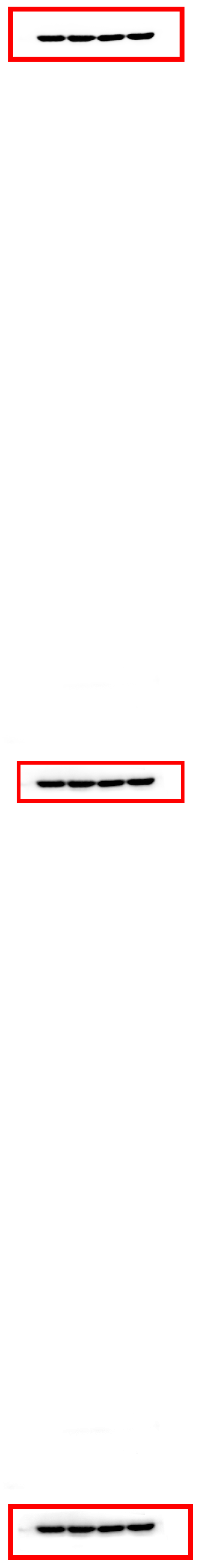

Fig4B Integrin α5

Three times repeated exposure image

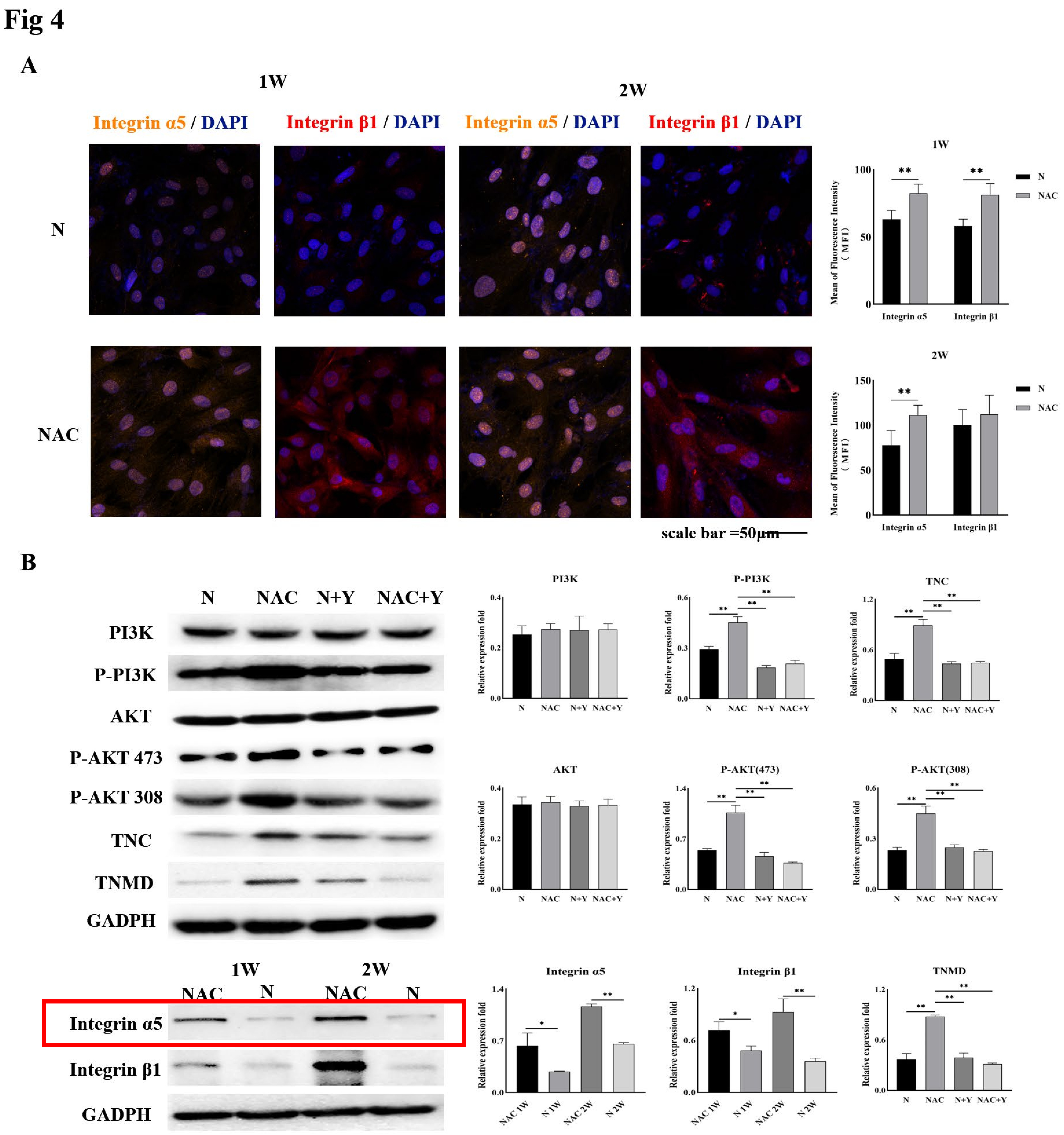

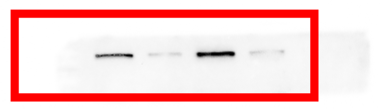

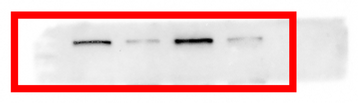

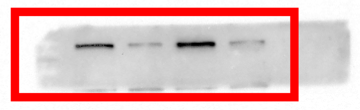

Fig4B Integrin β1

Three times repeated exposure image

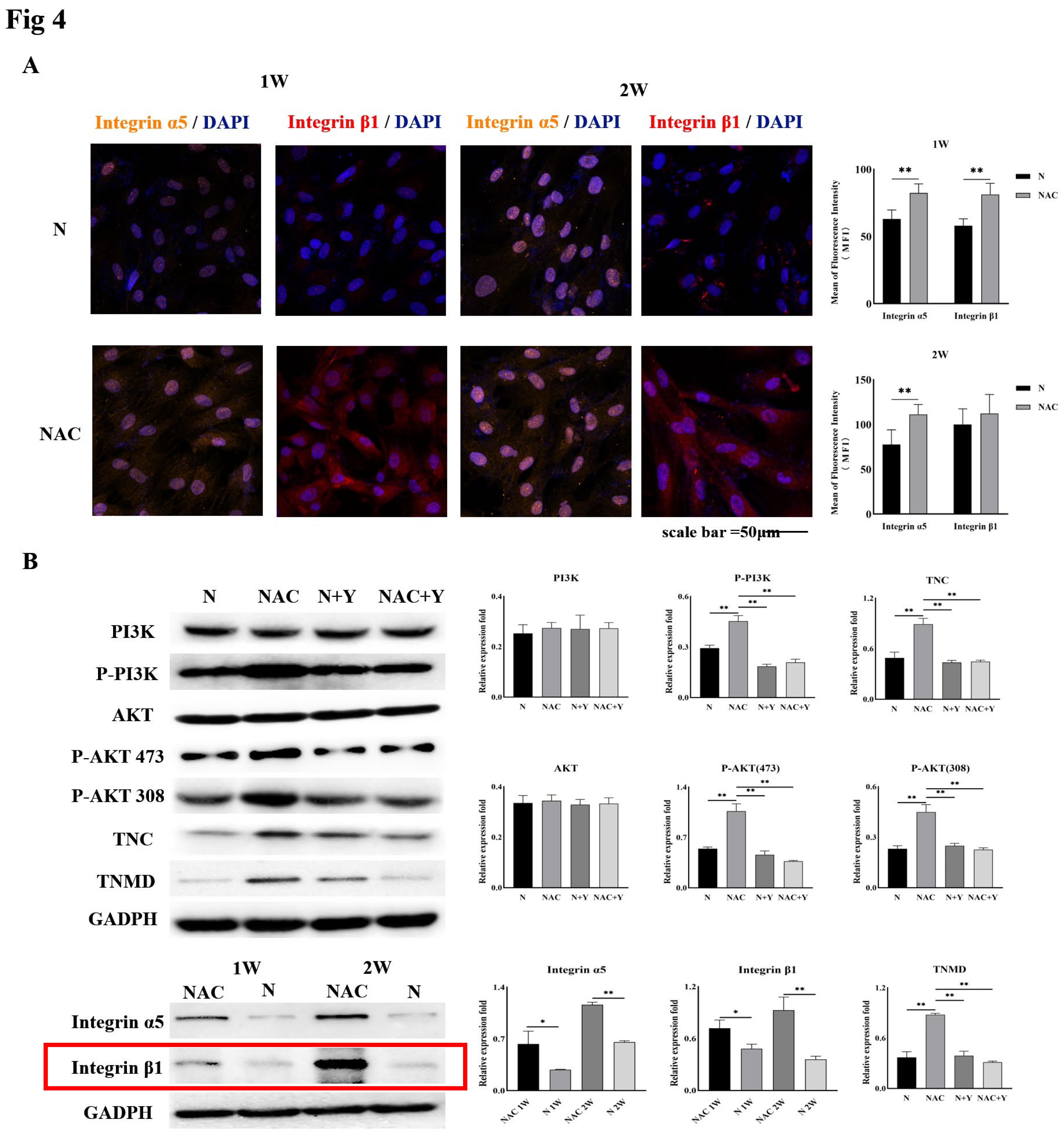

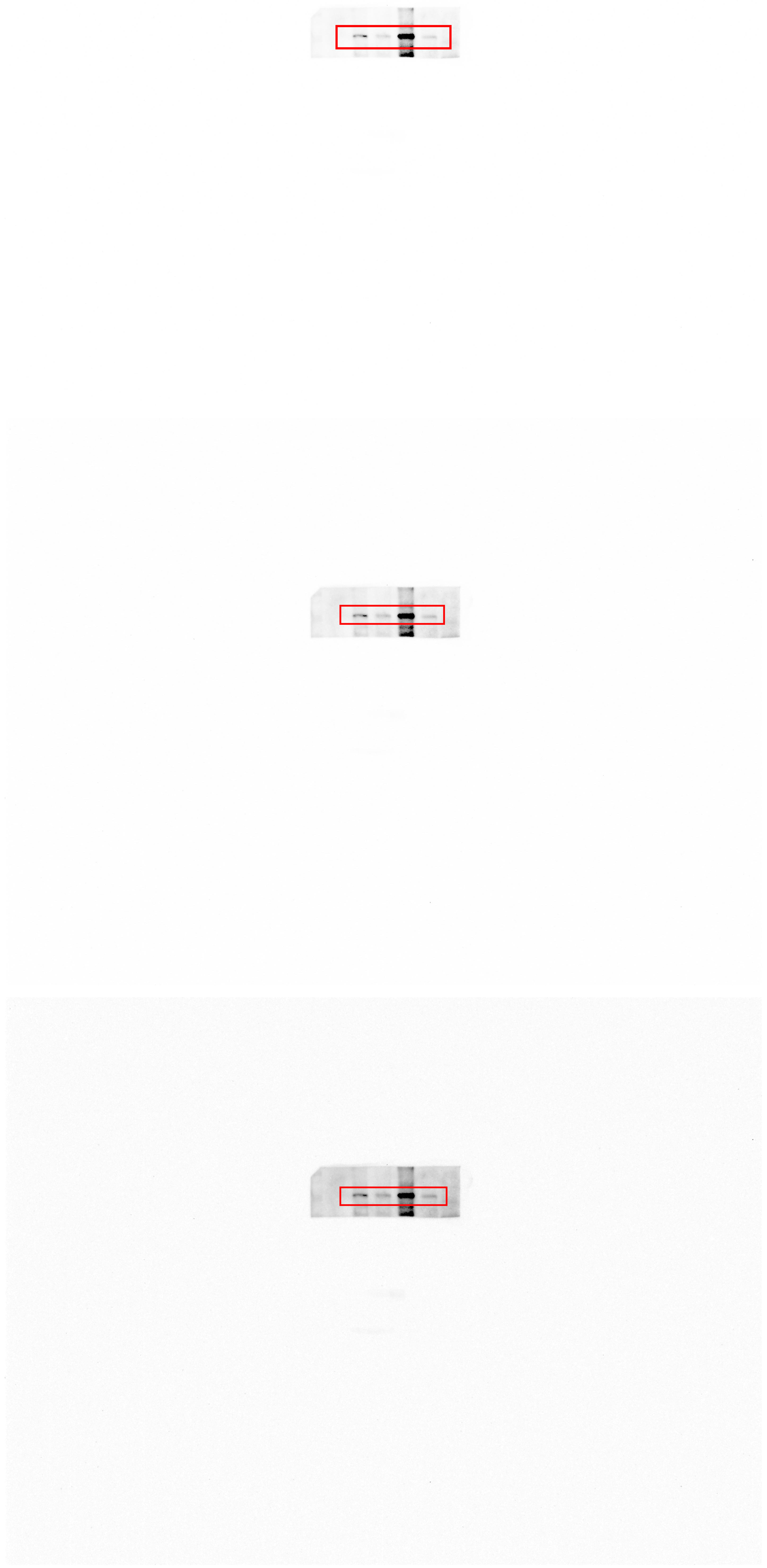

Fig4B Integrin-GADPH

Three times repeated exposure image

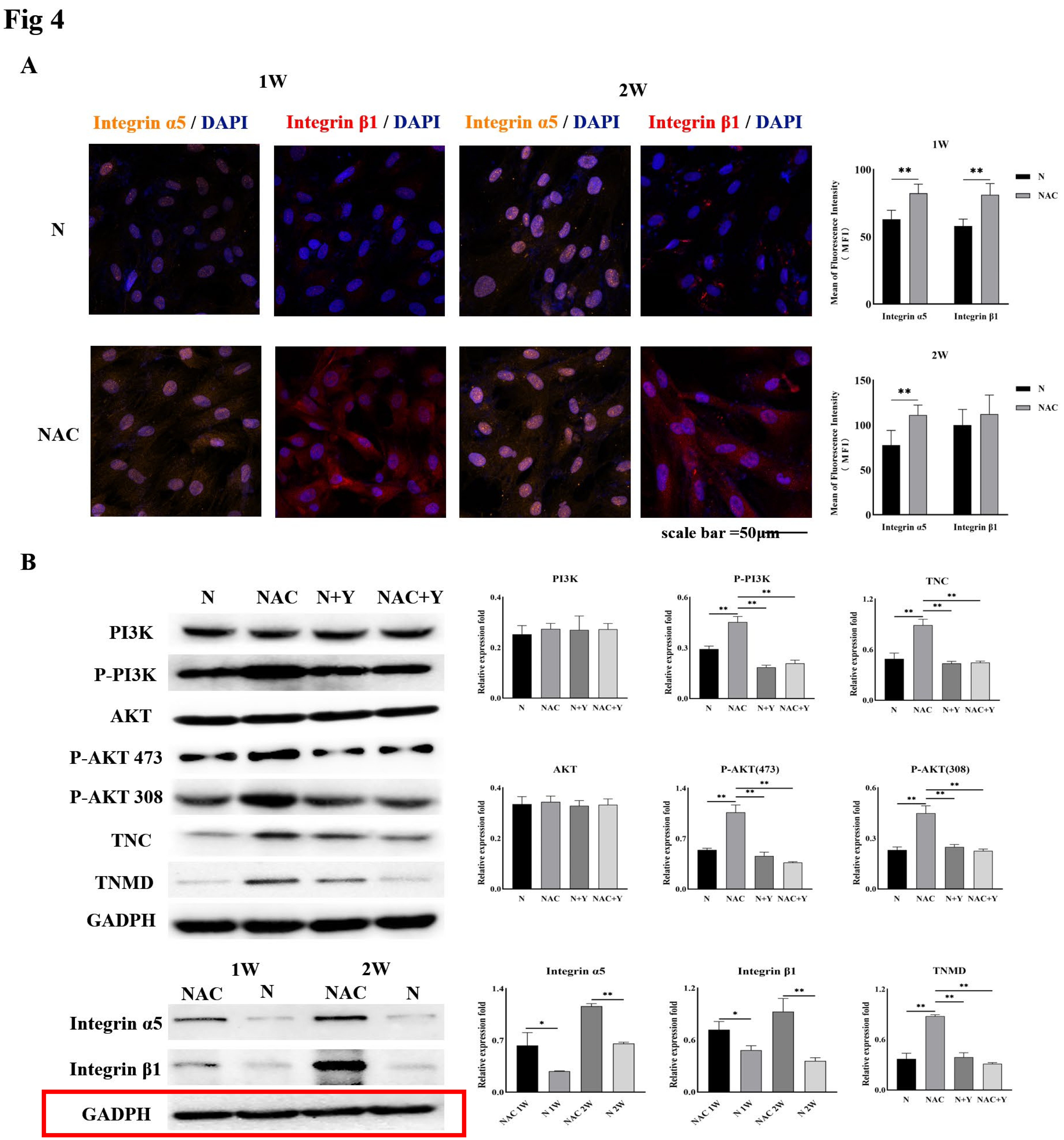

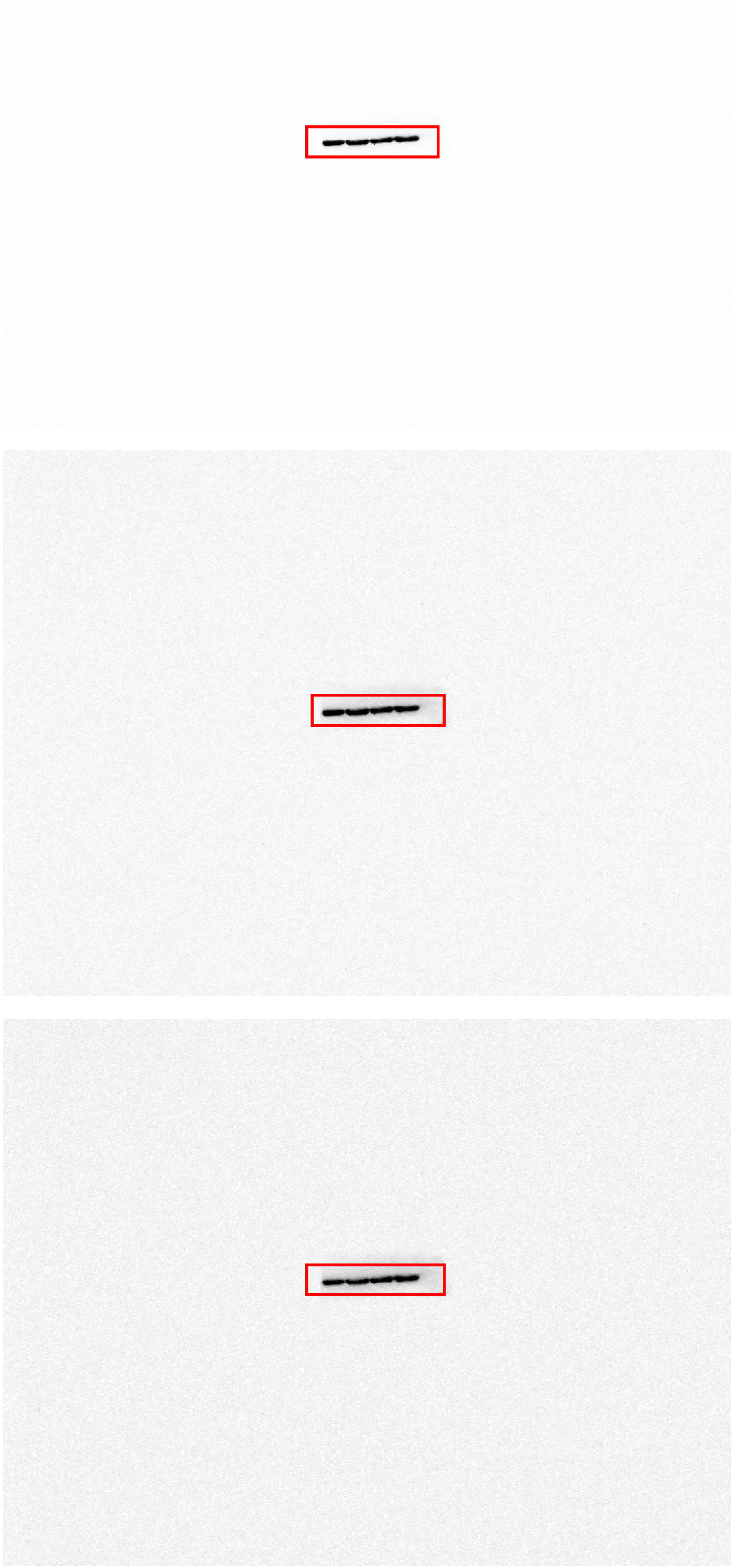

Supplement: Supplementary file 4 — Additional file 4. [file 12860_2022_463_MOESM4_ESM.pdf]
